# Identification of Novel Artemisinin Hybrids Induce Apoptosis and Ferroptosis in MCF-7 Cells

**DOI:** 10.3390/ijms232415768

**Published:** 2022-12-12

**Authors:** Ye Zhong, Zhi-Ning Li, Xin-Yue Jiang, Xing Tian, Ming-Hui Deng, Mao-Sheng Cheng, Hua-Li Yang, Yang Liu

**Affiliations:** Key Laboratory of Structure-Based Drug Design & Discovery, Ministry of Education, School of Pharmaceutical Engineering, Shenyang Pharmaceutical University, Shenyang 110016, China

**Keywords:** 1,3,4-oxadiazole, artemisinin, apoptosis, ferroptosis, antitumor

## Abstract

A series of novel 1,3,4-oxadiazole-artemisinin hybrids have been designed and synthesized. An MTT assay revealed that most of tested hybrids showed more enhanced anti-proliferative activities than artemisinin, among which A8 had the superior potency with IC_50_ values ranging from 4.07 μM to 9.71 μM against five tested cancer cell lines. Cell colony formation assays showed that A8 could inhibit significantly more cell proliferation than artemisinin and 5-fluorouracil. Further mechanism studies reveal that A8 induces apoptosis and ferroptosis in MCF-7 cells in a dose-dependent manner, and CYPs inhibition assays reveal that A8 has a moderate inhibitory effect on CYP1A2 and CYP3A4 in the human body at 10 μM. The present work indicates that hybrid A8 may merit further investigation as a potential therapeutic agent.

## 1. Introduction

Cancer, also known as malignant tumor, is a major disease that seriously threatens human health. In recent years, the incidence and death rates of cancer have increased due to many factors [1,2]. In 2021, it was estimated that there were 19.29 million new cancer patients and 9.96 million cancer deaths worldwide. By 2030, there will be more than 24.1 million new cancer patients and 13 million deaths worldwide [3]. Therefore, it is urgent to find effective clinical methods and new anticancer drugs for the prevention or treatment of cancer.

**Figure 1:**
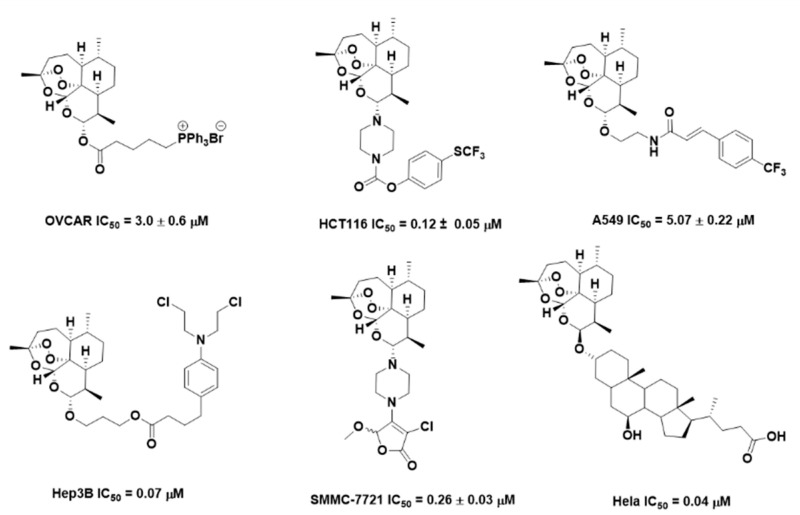
Successful modification of novel artemisinin derivatives or hybrids.

The extraction, separation and structural modification of active natural products is an ancient but effective method for drug discovery. Many drugs from natural products still have profound impacts on human beings [4,5,6,7]. Artemisinin (ART), an important naturally occurring endoperoxide-containing sesquiterpene lactone with excellent antimalarial activity, has also been proven to exhibit significant antitumor activity in recent research [8,9,10], and several successful structural modifications or hybrids of artemisinin have been reported [11,12,13,14,15,16] (Figure 1).

The hybrid strategy is a common design method in the development of new drug candidates. Compared with single pharmacophores, hybrid entities have many advantages, such as enhanced drug activity, improved pharmacokinetic properties, abrogated drug resistance and reduced toxicity and side effects [17,18,19,20]. Oxadiazoles, privileged moieties of nitrogen-based five-membered heterocycles, have been widely studied due to their broad range of pharmacological activities [21,22,23]. Among them, 1,3,4-oxadiazole has attracted the attention of researchers due to its unique pharmacokinetic properties and potential activities. Some 1,3,4-oxadiazole-containing hybrids have been proven to have good antitumor activity, as reviewed by Nayak et al. and Wang et al. [24,25].

Guided by the above information, a series of 1,3,4-oxadiazole-artemisinin hybrids have been designed and synthesized. Among them, hybrid **A8** was identified as an apoptosis and ferroptosis inducer in MCF-7 cells, and CYPs inhibition assays revealed that **A8** has no obvious inhibitory effects on the five major metabolic enzymes in the human body, suggesting that the risk of drug-drug interactions is very low and indicating that it could be used as a valuable lead compound for further study.

## 2. Results and Discussion

### 2.1. Chemistry

**Scheme 1:**
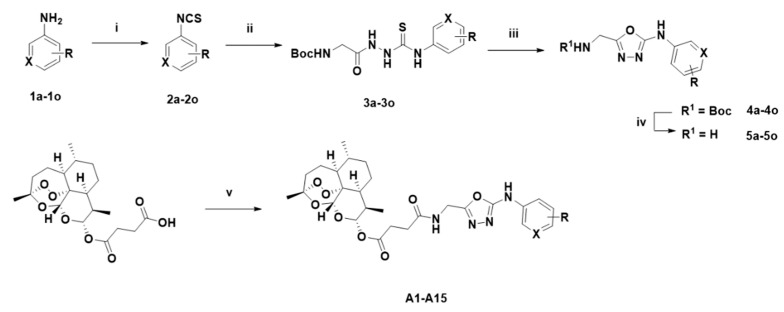
Reagents and conditions: (**i**) CSCl_2_, Et_3_N, dry THF, N_2_, 0 °C-r.t., 2 h; (**ii**) t-Butyl (2-hydrazinyl-2-oxoethyl)carbamate, dry THF, r.t., overnight; (**iii**) EDCI, DMSO, 60 °C, 5 h; (**iv**) 4M HCl/Diox, r.t., overnight; (**v**) EDCI, HOBt, DMF, r.t., 5 h.

The synthetic routes of target intermediates **2a**–**2o**, **3a**–**3o**, **4a**–**4o**, **5a**–**5o** and compounds **A1**–**A15** are illustrated in Scheme 1. Target intermediates **2a**–**2o** were prepared by commercially available aromatic amines and thiophosgene, and intermediates **2a**–**2o** were then added with t-butyl (2-hydrazinyl-2-oxoethyl)carbamate to form intermediates **3a**–**3o**, which were cyclized to give **4a**–**4o** with the help of EDCI [26]. The Boc moieties of **4a**–**4o** were removed by 4M HCl/Diox to form intermediates **5a**–**5o**, which were condensed with artesunate to give the target hybrids **A1**–**A15**. The correspondence between numbers and substituents is illustrated in Table 1 and Table S1.

### 2.2. In Vitro Anti-Cancer Activity Evaluation

The target hybrids **A1**–**A15** were screened for their antiproliferation activities against five human cancer cell lines (lung cancer A549 cells, breast cancer MCF-7 and MDA-MB-231 cells, colon cancer HCT116 cells and ovarian cancer SK-OV-3 cells) in vitro by the standard MTT method, and 5-fluorouracil (5-FU) and ART were coassayed as controls. The IC_50_ values are presented in Table 1. Notably, most of the synthesized hybrids showed more potent anti-proliferative activities than 5-FU and ART. In particular, hybrid **A8** exhibited better activities against all five tested cancer cell lines, with IC_50_ values of 7.7, 4.07, 9.71, 7.15 and 5.27 μM. Interestingly, more than half of the hybrids (**A1**–**A3**, **A8–A10**, **A12**–**A14**) exhibited strong inhibitory activities on MCF-7 cells with IC_50_ values of 4.07~11.14 μM, which revealed that MCF-7 cells may be more sensitive to this series of hybrids. Therefore, the most promising hybrid, **A8**, was chosen for further exploration of the preliminary mechanism of MCF-7 cell antiproliferation.

### 2.3. Cell Colony Formation Assay

**Figure 2:**
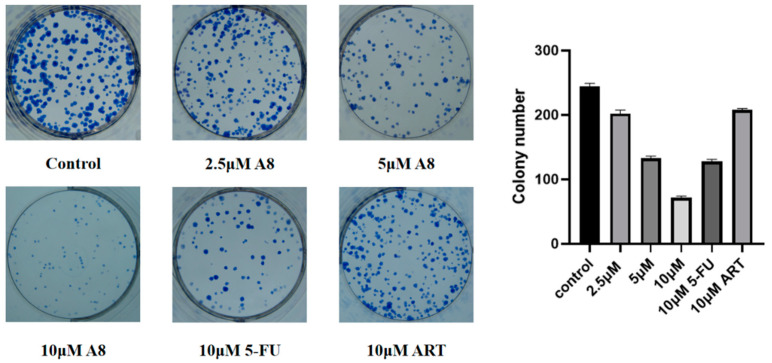
The effect of **A8**, 5-FU and ART towards MCF-7 cell colony number; 5-FU and ART were used as control.

We chose a cell colony formation assay to determine the antiproliferative effect of the compound more intuitively. As shown in Figure 2, the cell colony number decreased in a dose-dependent manner. Meanwhile, the antiproliferative effect of **A8** was better than that of 5-FU and ART at the same administration concentration. This result confirmed the strong in vitro antiproliferative effect of **A8**.

### 2.4. **A8** Induces Apoptosis in MCF-7 Cell Lines

#### 2.4.1. Analysis of Apoptosis by Annexin V-FITC/PI and TUNEL Staining

**Figure 3:**
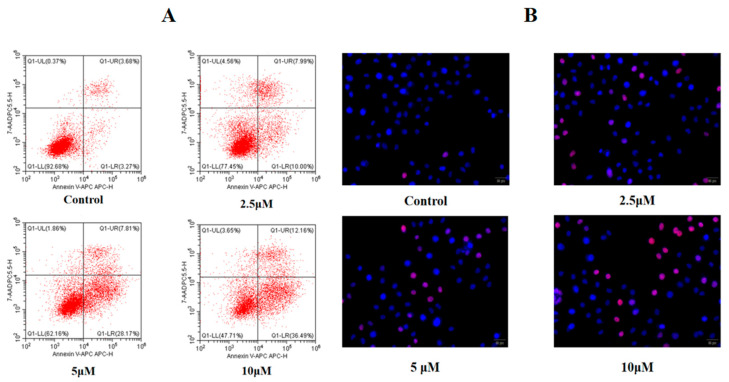
Apoptosis effects on MCF-7 cells induced by **A8**. (**A**) **A8**-induced apoptosis in MCF-7 cells. MCF-7 cells were incubated with varying concentrations of **A8**. After 48 h of incubation, cells were collected and stained with Annexin V/PI, followed by flow cytometric analysis. (**B**) Cell morphological alteration and nuclear change associated with MCF-7 cells after treatment with varying concentrations of **A8** were assessed by TUNEL staining and visualized by fluorescence microscopy. Bars denote 50 μm.

To investigate whether hybrid **A8** exerts an antitumor effect on MCF-7 cells by inducing apoptosis, an annexin V-FITC/PI binding assay was performed. MCF-7 cells were treated with vehicle or various concentrations (2.5, 5 and 10 μM) of hybrid **A8** for 48 h and were then stained with FITC-annexin V and propidium iodide (PI). The percentages of apoptotic MCF-7 cells were determined by flow cytometry. The results are shown in Figure 3A. **A8**-induced apoptosis in a dose-dependent manner. The percentages of total apoptotic cells (Q1-UR + Q1-LR) were 17.99% (2.5 μM), 35.98% (5 μM) and 48.65% (10 μM). To observe apoptosis more intuitively, TUNEL staining was also performed, and the results are shown in Figure 3B. The number of cells that emitted red fluorescence (apoptotic cells) increased as the administration concentration increased. In addition, obvious changes in cell morphology were also observed. These results demonstrated that **A8** could induce MCF-7 cell apoptosis.

#### 2.4.2. Analysis of Mitochondrial Membrane Potential (MMP) by JC-1 Staining

**Figure 4:**
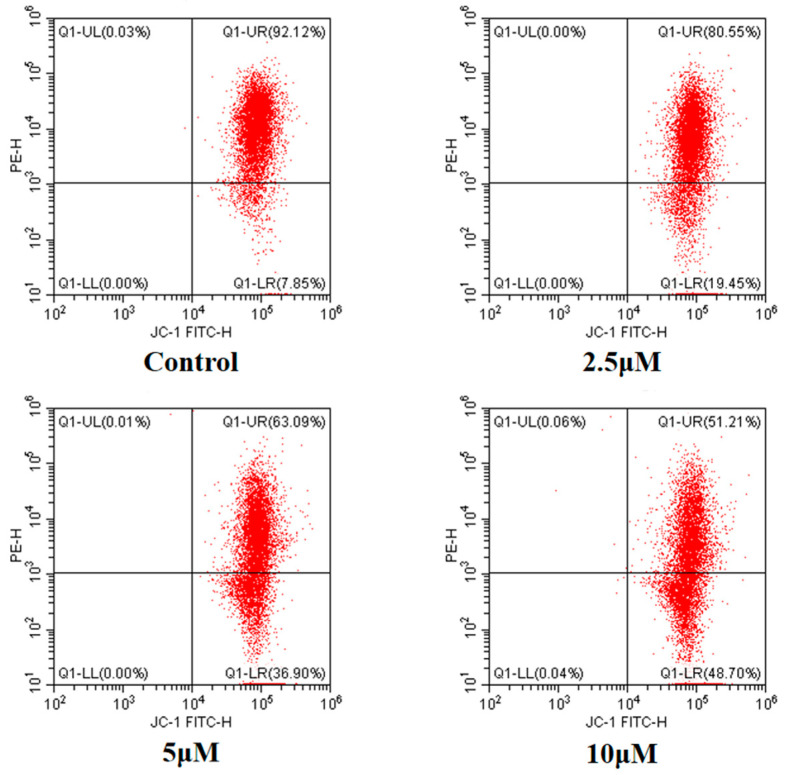
Effects of hybrid **A8** on MCF-7 cells MMP, MCF-7 cell were treated with compound **A8** at 0, 2.5, 5 and 10 μM for 48 h and stained with TMRE before flow cytometry analysis. Data were expressed as mean ± SD (n = 3). *p* < 0.05 versus the controls (0 μM).

JC-1 staining was also performed to explore the change in mitochondrial membrane potential (MMP), which could induce apoptosis. As shown in Figure 4, the flow cytometry studies revealed a concentration-dependent decrease in MMP after cells were treated with 0–10 μM **A8**, and the proportion of MCF-7 cells with depolarized mitochondria was increased (19.45%, 36.90% and 48.70%, respectively), compared to the control group (7.85%).

#### 2.4.3. Analysis of the Expression of Related Proteins by Western Blotting

**Figure 5:**
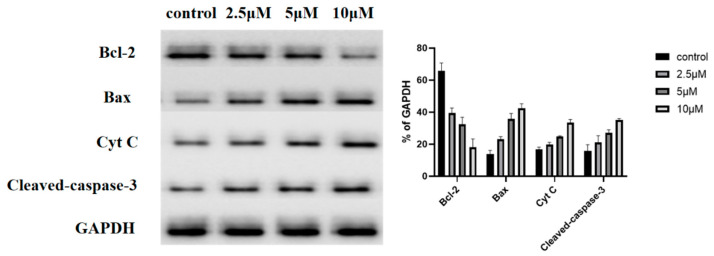
Effects of hybrid **A8** on the expression of related proteins of MCF-7 cells. Cells were treated with 0, 2.5, 5 and 10 μM of compound A8 for 48 h, and GAPDH was used as the internal control.

Based on the JC-1 staining results, we further analyzed the expression of proteins related to apoptosis using a Western blotting assay with GAPDH as an internal control. As shown in Figure 5, the expression levels of Bcl-2 and Bax, which are related to the mitochondrial membrane potential (MMP), were inversely correlated. Hybrid **A8** significantly downregulated the expression of Bcl-2 and upregulated the expression of Bax, which caused the mitochondrial membrane potential to decrease and cytochrome C to be released. Then, an excess of cytochrome C can enhance caspase-3 activation to stimulate apoptosis. Above all, the loss of MMP and the results of the Western blotting assay indicated that **A8** could induce MCF-7 cell apoptosis via the mitochondrial apoptotic pathway.

### 2.5. **A8** Induces Ferroptosis in MCF-7 Cells

Ferroptosis, an iron-dependent, nonapoptotic form of regulated cell death that is independent of caspase activation, is often accompanied by the accumulation of reactive oxygen species (ROS) and lipid peroxides, a decrease in reduced glutathione (GSH) levels and the downregulation of glutathione peroxidase 4 (GPX4) [27,28]. Recent studies indicate that the peroxide bridge of ART could destroy the redox balance in tumor cells, resulting in oxidative stress and ROS accumulation [10,29]. Thus, we speculated that ROS-induced ferroptosis also occurred. All of these were, therefore, quite reasonable options for us to use to study the ferroptosis pathway of **A8**-induced MCF-7 cell death.

#### 2.5.1. **A8** Induces ROS Generation and Accumulation

**Figure 6:**
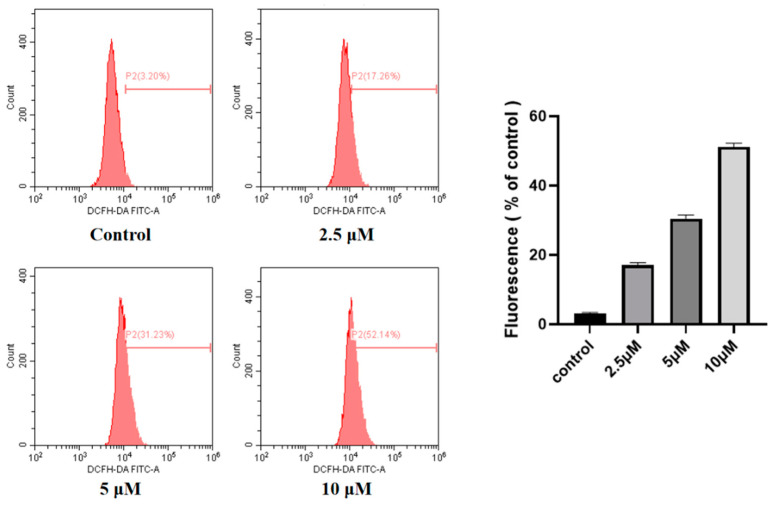
Effects of hybrid **A8** on the ROS level of MCF-7 cells. MCF-7 cells treated with **A8** at the concentrations of 0, 2.5, 5 and 10 μM for 48 h and loaded with DCFH-DA fluorescent probe before analysis.

First, we detected ROS levels using DFCH-DA. As shown in Figure 6, the fluorescence intensity increased significantly with increasing drug concentration, which suggested that hybrid **A8** could induce ROS generation and accumulation in MCF-7 cells in a dose-dependent manner.

#### 2.5.2. **A8** Induces the Downregulation of GSH Expression and Lipid Peroxide Accumulation

**Figure 7:**
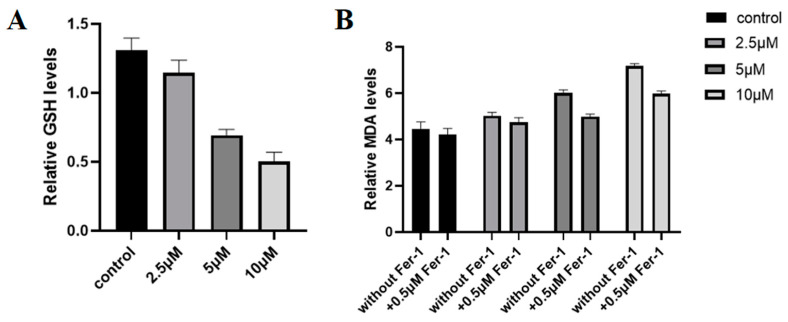
Effects of hybrid **A8** on the GSH level (**A**) and MDA level (**B**) of MCF-7 cells; Micro Reduced Glutathione (GSH) Assay Kit was used for GSH detection and Lipid Peroxidation MDA Assay Kit was used for measuring MDA level.

We also determined the effect of **A8** on the expression of GSH and MDA (lipid peroxidation). As shown in Figure 7, the GSH level decreased significantly, while the MDA level increased at higher concentrations, and the addition of ferrostatin-1 (Fer-1, a ferroptosis inhibitor) reversed lipid peroxide accumulation, which revealed that **A8** could induce ferroptosis-related downregulation of GSH expression and lipid peroxide accumulation.

#### 2.5.3. **A8** Induces the Downregulation of GPX4 Expression

**Figure 8:**
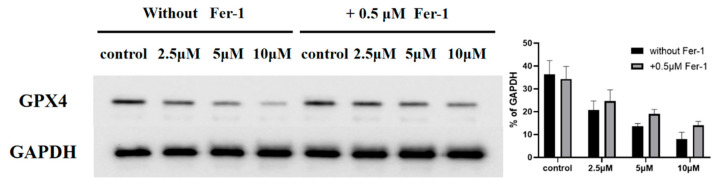
Effects of hybrid **A8** on the expression of GPX4 in MCF-7 cells; cells were treated with 0, 2.5, 5 and 10 μM of compound **A8** for 48 h, and GAPDH was used as the internal control.

We examined the effect of **A8** on the expression of GPX4 as well, which is vital in preventing ferroptosis. As shown in Figure 8, a dose-dependent inhibition of GPX4 expression was induced by **A8**, while the addition of Fer-1 restored the expression of GPX4. Thus, we concluded that **A8** induced the accumulation of ROS and lipid peroxidation and suppressed GSH levels and the expression of GPX4, thereby leading to the induction of ferroptosis.

### 2.6. CYPs Inhibition Assay

Finally, we evaluated the interaction between the hybrid **A8** and five major metabolic enzymes (CYP1A2, CYP2C9, CYP2C19, CYP2D6 and CYP3A4) in the human body. The result were shown in Table 2, CYPs inhibition studies showed that hybrid **A8** exhibits a moderate inhibitory effect on CYP1A2 and CYP3A4 at 10 μM concentration, which suggested a potent drug–drug interaction with other clinical drugs. It is also one of the directions for our further transformation and optimization.

## 3. Materials and Methods

### 3.1. Chemistry

All the reagents (Energy Chemical, Shanghai, China) were used without further purification unless otherwise specified. Solvents were dried and redistilled prior to use in the usual manner. Analytical TLC was performed using silica gel HF254 (Qingdao Haiyang Chemical, Qingdao, Shandong, China). Preparative column chromatography was performed with silica gel H. Melting points were obtained on a Büchi melting point B-540 apparatus (Büchi Labortechnik, Flawil, Switzerland). ^1^H and ^13^C NMR spectra were recorded on a Bruker ARX 600 MHz spectrometer (Bruker, Zurich, Switzerland); ESI-MS were obtained by an Agilent ESI-LC-MSD instrument (Agilent, Santa Clara, CA, USA). The synthetic routes and NMR spectra are shown in Supporting Information (Figure S1–S46).

### 3.2. Pharmacology

#### 3.2.1. Cell Cytotoxicity Assay

Cytotoxicity of test compounds against five different tumor cell lines were evaluated using an MTT assay in vitro. Cells were seeded into 96-well plates at a density of 5 × 10^4^ cells per well and stabilized at 37 °C with 5% CO_2_ for 24 h. Compounds **A1–A15**, ART and 5-FU were added to each well at various concentrations and then the cells were incubated for 48 h. The MTT solution (100 μL 0.5 mg/mL^−1^) was added to each well, and the cells were incubated for another 4 h. Then, 150 μL DMSO was added to each well and the absorbance of samples was measured at 492 nm. The IC_50_ values were calculated according to Logit method after getting the inhibitory rate.

#### 3.2.2. Cell Colony Formation Assay

MCF-7 cells were seeded into 6-well plates at 37 °C with 5% CO_2_ for 24 h and then treated with compound **A8** at various concentrations (0, 2.5, 5 and 10 μM), ART (10 μM) and 5-FU (10 μM) for 12 d. Then, cells were stained with Giemsa and clones number were counted directly with naked eyes.

#### 3.2.3. Annexin V/PI Staining Assay

Cells apoptosis was assessed using Annexin V/PI staining assay. MCF-7 cells were seeded into 6-well plates for 24 h and then treated with compound **A8** at various concentrations (0, 2.5, 5 and 10 μM) for 48 h. Then, cells were collected, washed with 500 μL annexin-binding buffer and stained with 5 μL annexin V-FITC and 5 μL PI for 15 min at 25 °C. After that, the samples were analyzed by flow cytometry (Beckman Coulter cytoFLEX, Brea, CA, USA).

#### 3.2.4. TUNEL Staining

The slides were immersed in 4% paraformaldehyde (pH 7.4) for 25 min at room temperature and then washed with PBS 3 times. The cells were immersed in 0.1% Triton X-100 solution prepared with PBS for 10 min (operation on ice) and then washed twice with PBS. Dilute 5 × equilibration buffer with deionized water and 100 μL 1 × equilibration buffer was added to each climbing tablet to cover the sample area to be tested, then incubated at room temperature for 15 min. After the buffer solution of 1 μD was added to the buffer solution, most of the buffer solution was added to the buffer solution of 1 μD, and then the buffer solution was used to wash off the buffer. The slides were placed in a wet box and incubated at 37 °C for 60 min. The wet box was wrapped with aluminum foil to avoid light. They were then washed 3 times with PBS with DAPI dripped and incubated in dark for 5 min. The specimens were stained with nuclei. The water-absorbent paper was used to absorb the liquid on the climbing sheet, and the sealing liquid containing anti fluorescence quenching agent was used to seal the film, and then the images were observed and collected under the fluorescence microscope.

#### 3.2.5. JC-1 Mitochondrial Membrane Potential Assay

MCF-7 cells were plated at 1 × 10^6^ cells per well in 24-well plates and incubated with compound **A8** at various concentrations (0, 2.5, 5 and 10 μM) for 48 h. Subsequently, the cells were incubated with JC-1 dye and finally analyzed by flow cytometry (Beckman Coulter cytoFLEX, USA).

#### 3.2.6. Intracellular ROS Generation

ROS production was detected by using the peroxide-sensitive fluorescent probe DCFH-DA. After treatment with different concentrations of **A8** for 48 h, the cells were incubated with 10 μg/mL DCFH-DA at 37 °C for 20 min. Then, cells were harvested. Samples were analyzed by flow cytometry (FACS Calibur, Becton-Dickinson, East Rutherford, NJ, USA).

#### 3.2.7. Lipid Peroxidation and GSH Level Detection

Lipid peroxidation assay kit (Beyotime, Shanghai, China) was used to detect malonaldehyde (MDA), together with GSH level assay, present in samples, as per the manufacturer’s instruction with slight modifications.

#### 3.2.8. Western Blotting Assay

MCF-7 cells were seeded at a density of 4 × 105 cells per well and treated with various concentrations of compound **A8** (0, 2.5, 5 and 10 μM) for 48 h. After this, cells were collected and washed twice with ice-cold DPBS. The pellets were resuspended in a total protein extraction buffer (20 mM HEPES, 350 mM NaCl, 20% glycerol, 1% NP-40, 1 mM MgCl_2_, 0.5 mM EDTA, 0.1 mM EGTA, 0.1 mM DTT, 0.1 mM PMSF) containing a protease inhibitor cocktail and incubated on ice for 30 min with intermittent mixing. The protein concentration was measured using the Bradford reagent (Bio-Rad Laboratories Inc., Hercules, CA, USA). An equal amount (20 μg) of protein was loaded on 10% polyacrylamide gels and transferred to a nitrocellulose membrane. After blocking with 5% skimmed milk, the membrane was incubated at 4 °C overnight with specific primary antibodies. The membrane was washed and incubated at room temperature for 1 h with secondary antibodies conjugated with horseradish peroxidase (HRP). Finally, the immunoblot was developed for visualization using a chemiluminescence kit. Primary antibodies for cytochrome C, cleaved-caspase-3, Bax, Bcl-2, GPX4 and GAPDH and all secondary antibodies were obtained from Santa Cruz Biotechnology (Santa Cruz, CA, USA).

#### 3.2.9. CYPs Inhibition Assay

Cytochrome P450 inhibition was evaluated in human liver microsomes (0.253 mg/mL) using five probe substrates (CYP1A2, α-Naphthoflavone; CYP2C9, Sulfaphenazole; CYP2C19, Benzylnirvanol; CYP2D6, Quinidine; and CYP3A4, Ketoconazole.) in the presence of compound **A8** (10 μM). After preincubation for 10 min at 37 °C, an NADPH-regenerating system was added. After the mixed system was incubated for 15 min at 37 °C, the reaction was stopped by adding 400 μL of cold stop solution (200 ng/mL tolbutamide and 200 ng/mL labetalol in acetonitrile). Then, the mixture was centrifuged, and the supernatant was analyzed by LC-MS/MS.

#### 3.2.10. Statistical Analysis

Values are presented as means ± SEM from at least three independent experiments, with a *p* value of less than 0.05 being considered statistically significant. Statistical analyses were performed using GraphPad Prism 8. Flow-cytometry-related experiment was analyzed by C Flow Plus. Cell colony formation assay and Western blotting were measured by Image J(x64) 2019.

## 4. Conclusions

In the present work, a series of novel 1,3,4-oxadiazole-artemisinin hybrids were designed and synthesized. The results of in vitro antiproliferation activities against five human cancer cell lines showed that most of the novel hybrids exhibit better antiproliferation effects than the control, and MCF-7 cell lines are more sensitive to this series of hybrids. Cell colony assays proved that the most promising hybrid, **A8**, could effectively inhibit the proliferation of MCF-7 cells. Further research on the mechanism revealed that A8 could induce apoptosis mediated by the mitochondrial pathway and ferroptosis in a concentration-dependent manner in MCF-7 cells. Moreover, the CYPs inhibition assay proved that **A8** has a moderate inhibitory effect on CYP1A2 and CYP3A4 in the human body at 10 μM concentration. These results provide good inspiration for the discovery of novel antitumor agents from artemisinin scaffolds, as well as new insight into the design of artemisinin-related hybrids, to enhance the efficacy of candidates.

## Figures and Tables

**Table 1:** IC_50_ values ^a^ (μM) of hybrid **A1**–**A15**, ART and 5-FU against five human cancer cell lines.

Com.	R	X	A549	MCF-7	HCT116	SK-OV-3	MDA-MB-231
**A1**	4-OCF_3_	C	>30	5.20 ± 1.38	>30	>30	>30
**A2**	4-CH_3_	C	>30	4.26 ± 0.17	14.85 ± 1.77	>30	4.89 ± 1.58
**A3**	2-OEt	C	>30	6.82 ± 0.25	>30	>30	>30
**A4**	2,6-*di*-F	C	>30	>30	>30	>30	>30
**A5**	4-CF_3_	C	>30	>30	>30	>30	>30
**A6**	4-Cl	C	>30	>30	>30	>30	>30
**A7**	2,5-*di*-Br	C	>30	>30	>30	>30	>30
**A8**	2,4-*di*-F	C	7.7 ± 0.36	4.07 ± 0.05	9.71 ± 0.37	7.15 ± 0.95	5.27 ± 0.02
**A9**	2-F	C	>30	8.89 ± 0.39	>30	>30	>30
**A10**	4-*t*-Bu	C	>30	11.14 ± 1.28	>30	>30	>30
**A11**	4-Br	C	>30	>30	>30	>30	>30
**A12**	H	N	>30	6.49 ± 0.79	>30	6.22 ± 0.72	>30
**A13**	3,5-*di*-CH_3_	C	>30	9.72 ± 0.11	>30	>30	>30
**A14**	4-OCH_3_	C	>30	6.86 ± 0.73	>30	>30	19.36 ± 0.91
**A15**	2,4-*di*-CH_3_	C	>30	>30	>30	>30	>30
ART	/	/	27.25 ± 0.67	>30	>30	>30	>30
5FU ^b^	/	/	>30	26.14 ± 0.87	18.38 ± 1.14	>30	27.67 ± 1.23

^a^ MTT methods, cells were incubated with corresponding compounds for 48 h. IC_50_ value (means ± SD, n = 3). ^b^ 5-FU as the positive control.

**Table 2:** Cytochrome P450 inhibition of hybrid **A8**.

Isozyme	CYP1A2	CYP2C9	CYP2C19	CYP2D6	CYP3A4
Inhibition at 10 μM (%)	57.1	6.4	12.3	0.1	55.8

## Data Availability

Not applicable.

## Supplementary Materials

The supporting information can be downloaded at: https://www.mdpi.com/article/10.3390/ijms232415768/s1.
