# Supplementary material for: Identification of Novel Artemisinin Hybrids Induce Apoptosis and Ferroptosis in MCF-7 Cells
Source: Int J Mol Sci. 2022 Dec 12;23(24):15768. doi: 10.3390/ijms232415768 (PMC9779727; doi:10.3390/ijms232415768)
Supplement: Supplementary file 1 [file ijms-23-15768-s001.zip › ijms-1959734-supplementary.pdf]

Supporting Information

Identification of novel artemisinin hybrids induce apoptosis and ferroptosis in MCF-7 cells

Ye Zhong, Zhi-Ning Li, Xin-Yue Jiang, Xing Tian, Ming-Hui Deng, Mao-Sheng Cheng, Hua-Li Yang * and Yang Liu *

Key Laboratory of Structure-Based Drug Design & Discovery of Ministry of Education, School of Pharmaceutical Engineering, Shenyang Pharmaceutical University, Shenyang 110016, China

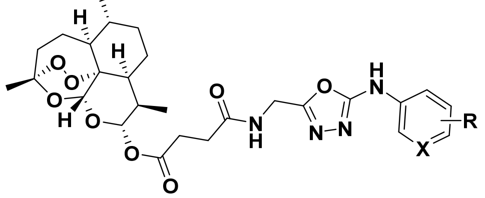

Table S1. list of hybrids and intermediates with various substituents

Num.	R	X
**1a-5a, A1**	4-OCF_3	C
**1b-5b, A2**	4-CH_3	C
**1c-5c, A3**	2-OEt	C
**1d-5d, A4**	2,6-2F	C
**1e-5e, A5**	4-CF_3	C
**1f-5f, A6**	4-Cl	C
**1g-5g, A7**	2,5-2Br	C
**1h-5h, A8**	2,4-2F	C
**1i-5i, A9**	2-F	C
**1j-5j, A10**	4-*t*-Bu	C
**1k-5k, A11**	4-Br	C
**1l-5l, A12**	H	N
**1m-5m, A13**	3,5-2CH_3	C
**1n-5n, A14**	4-OCH_3	C
**1o-5o, A15**	2,4-2CH_3	C

^{1}H-NMR And ^{13}C-NMR Spectrum

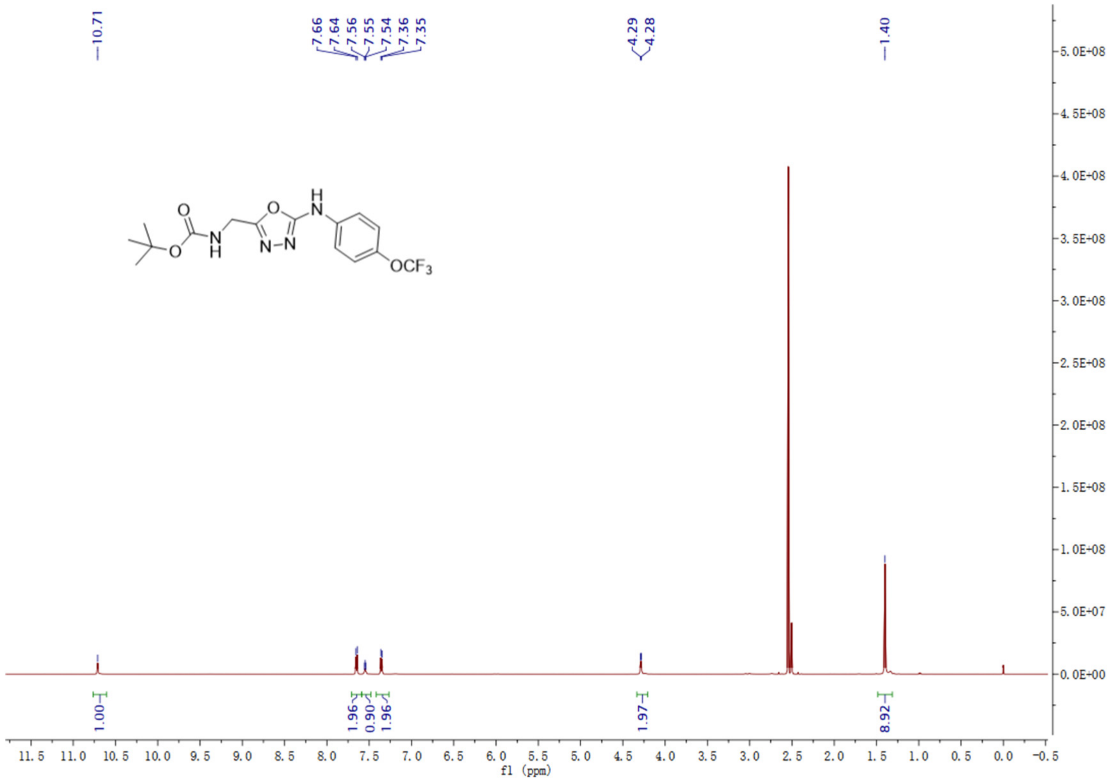

Figure S1. ^{1}H-NMR spectrum of Intermediate **4a**

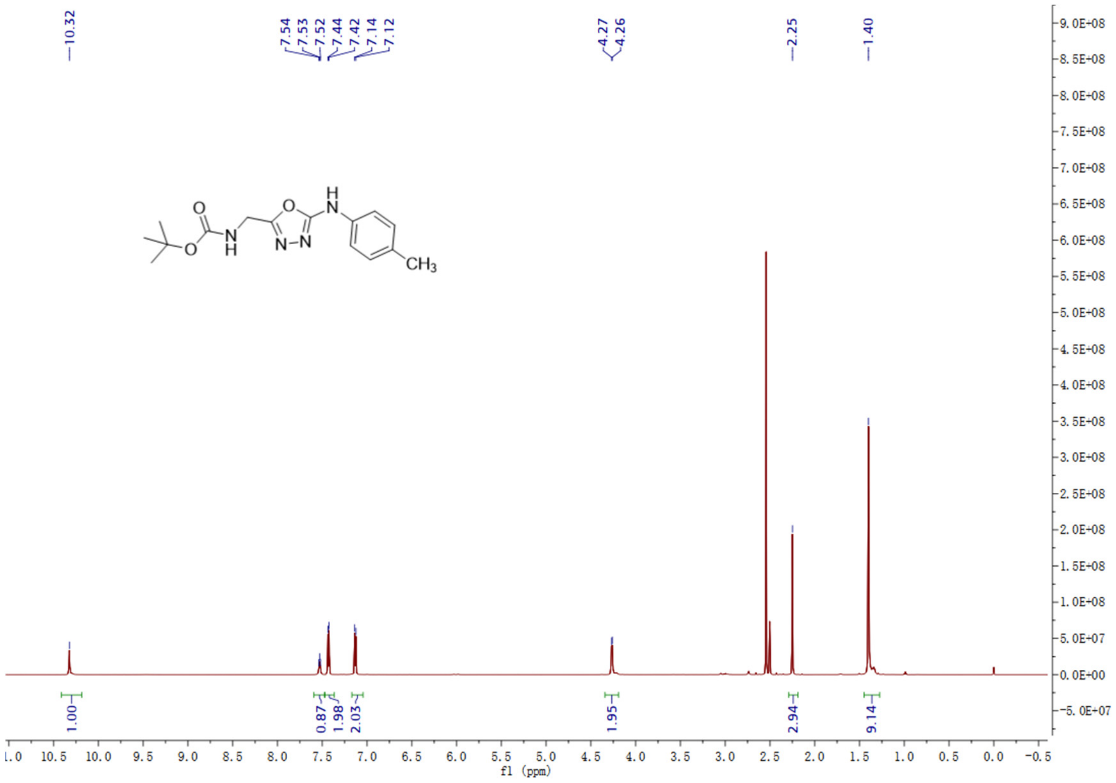

Figure S2. ^{1}H-NMR spectrum of Intermediate **4b**

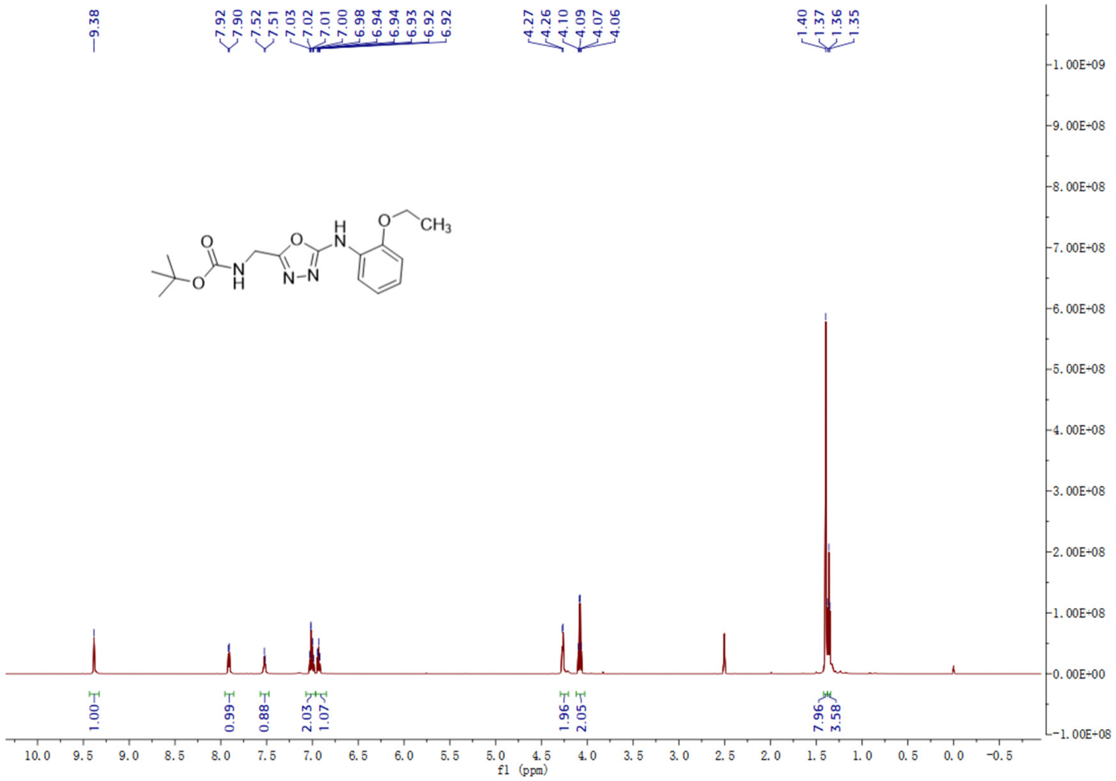

Figure S3. ^{1}H-NMR spectrum of Intermediate **4c**

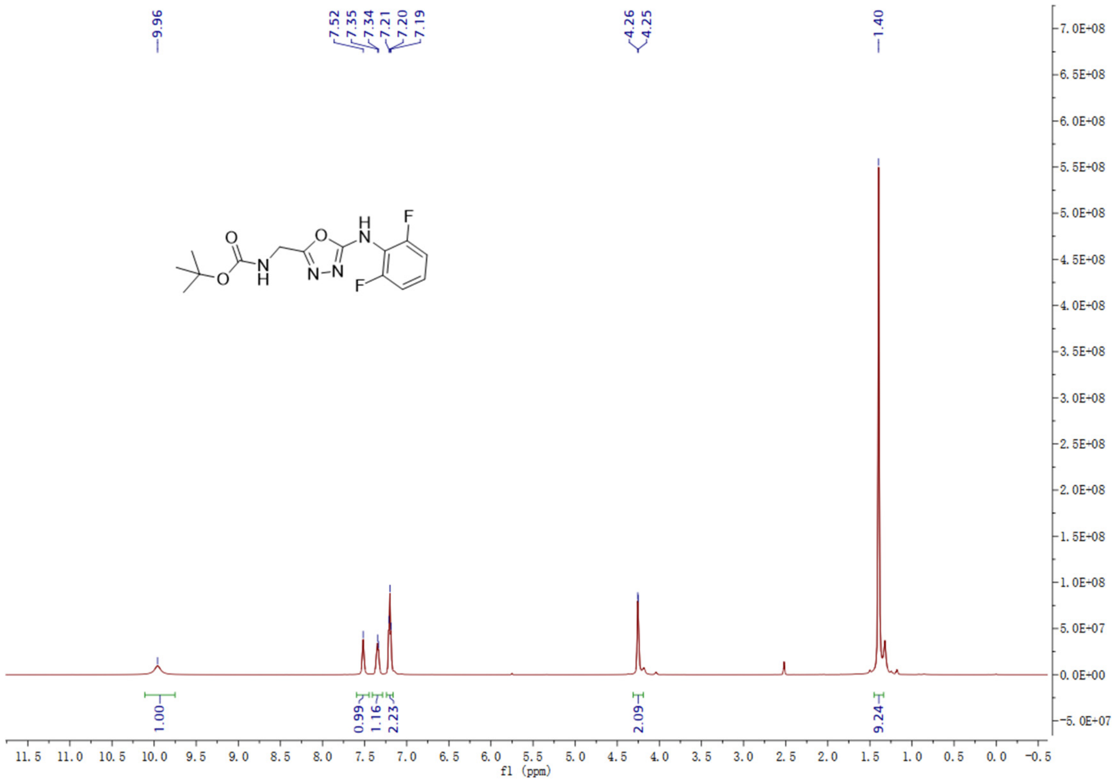

Figure S4. ^{1}H-NMR spectrum of Intermediate **4d**

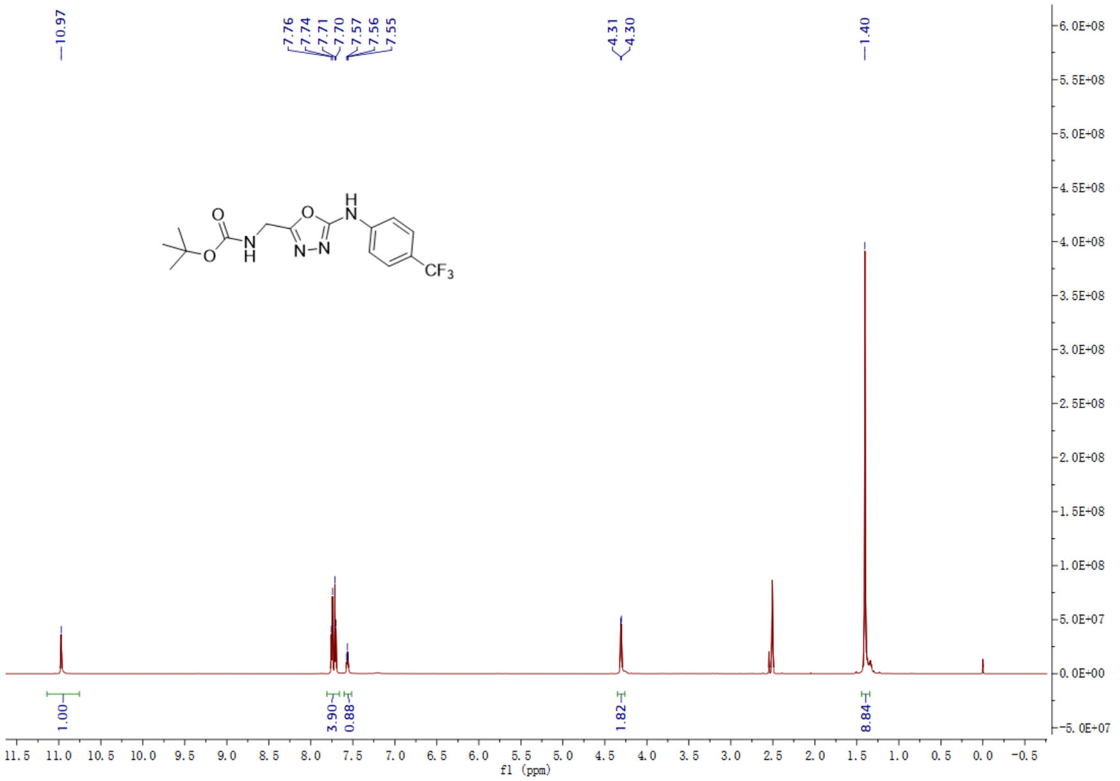

Figure S5. ^{1}H-NMR spectrum of Intermediate **4e**

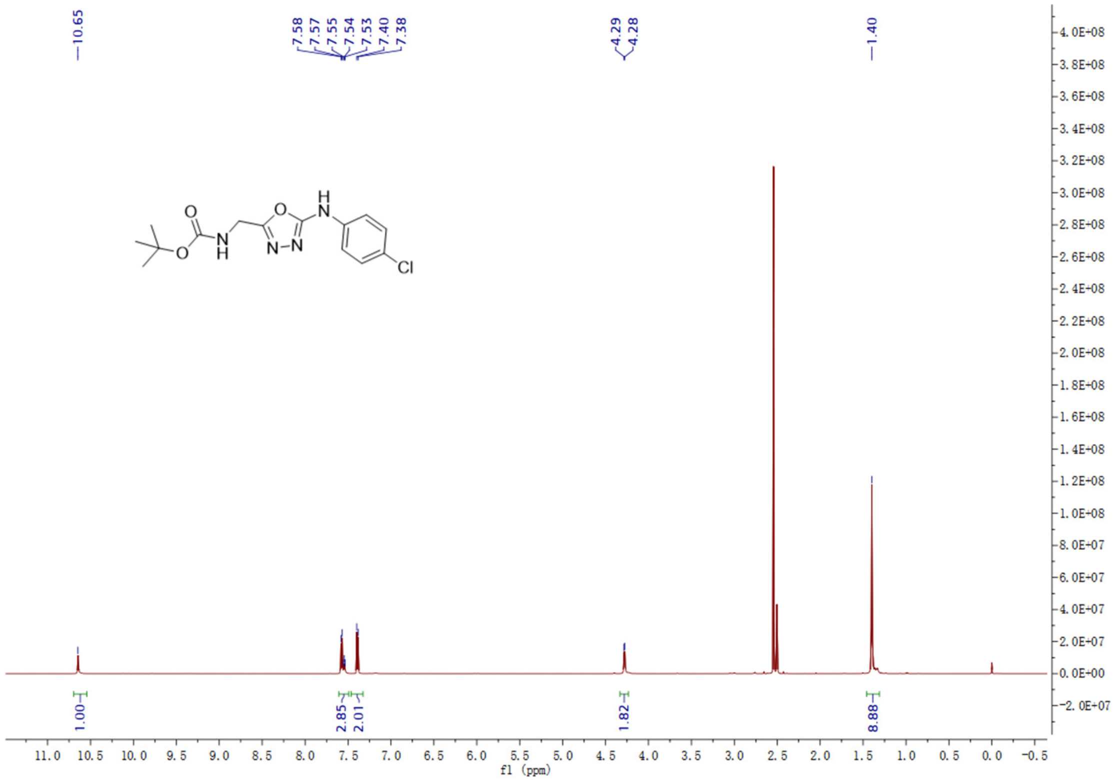

Figure S6. ^{1}H-NMR spectrum of Intermediate **4f**

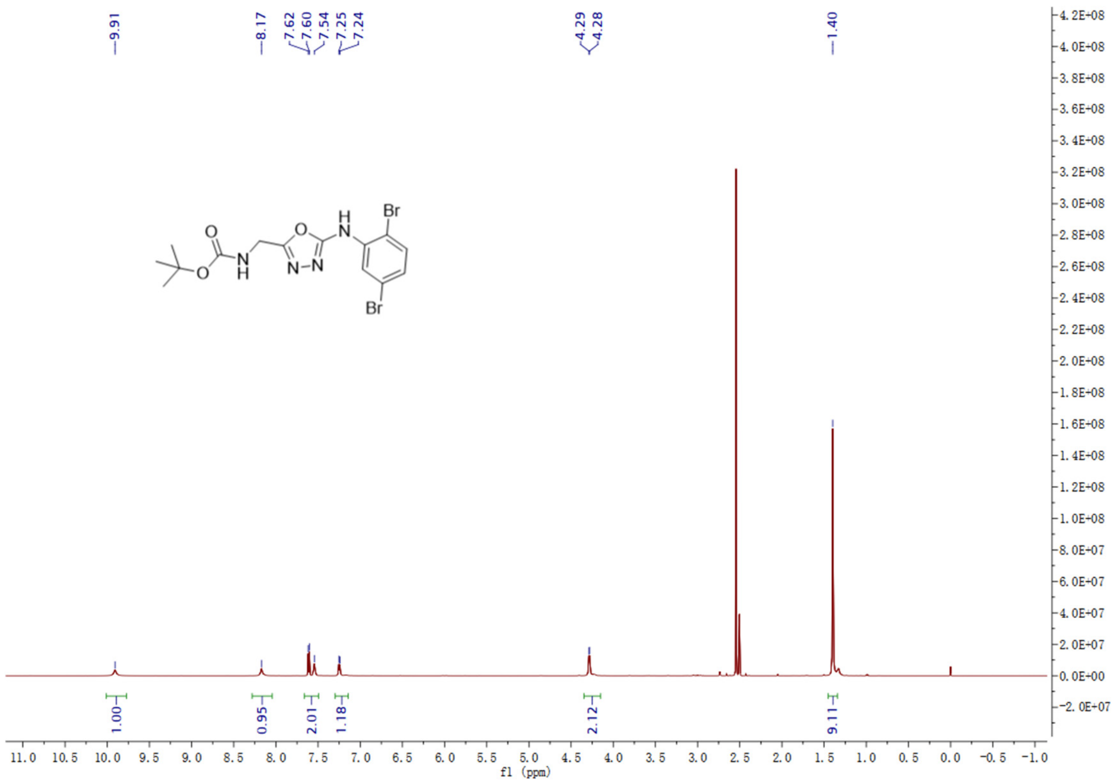

Figure S7. ^{1}H-NMR spectrum of Intermediate **4g**

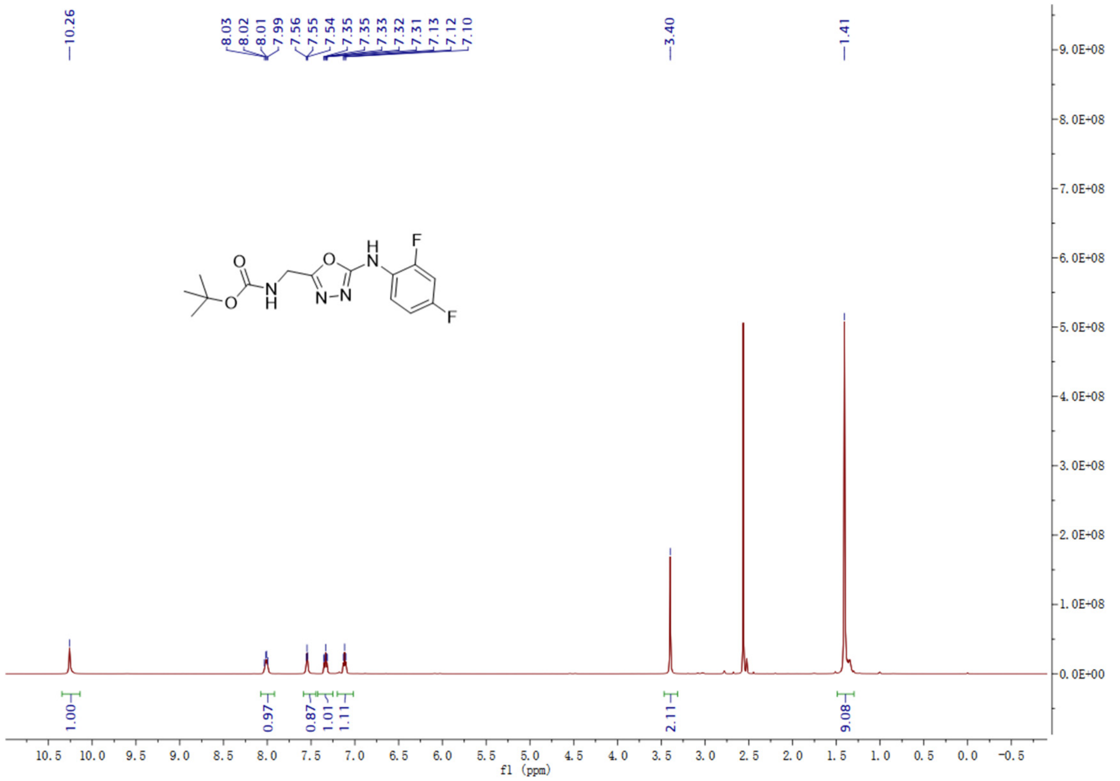

Figure S8. ^{1}H-NMR spectrum of Intermediate **4h**

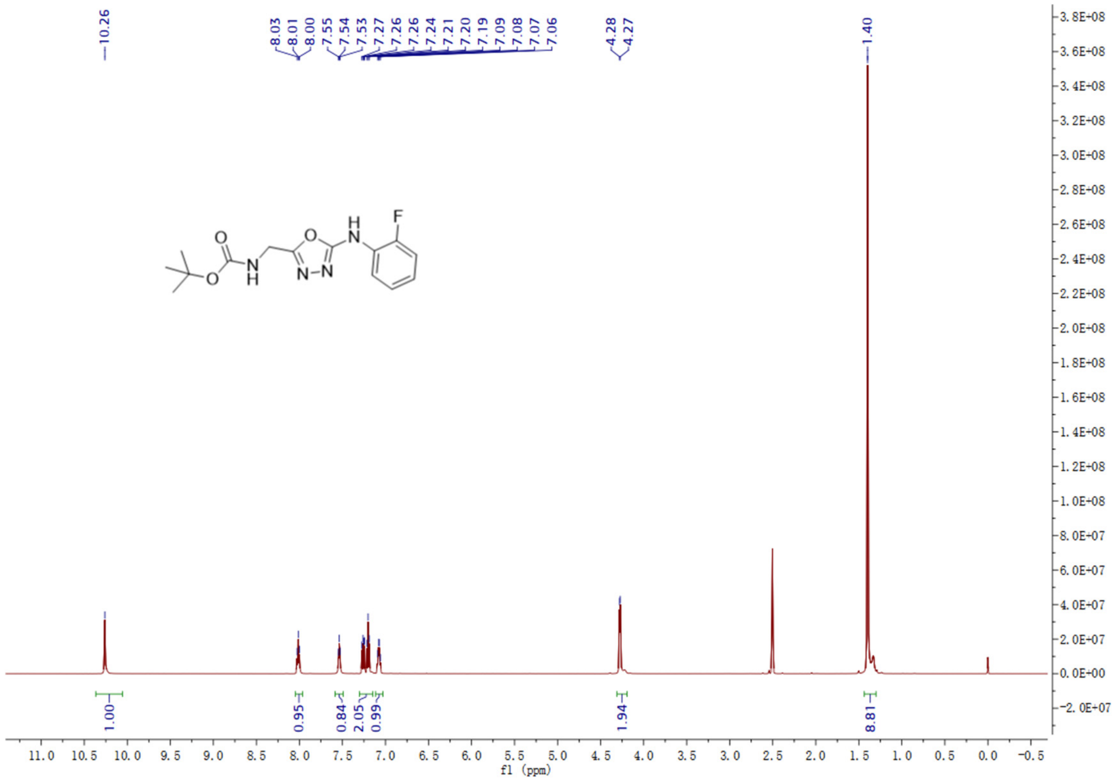

Figure S9. ^{1}H-NMR spectrum of Intermediate **4i**

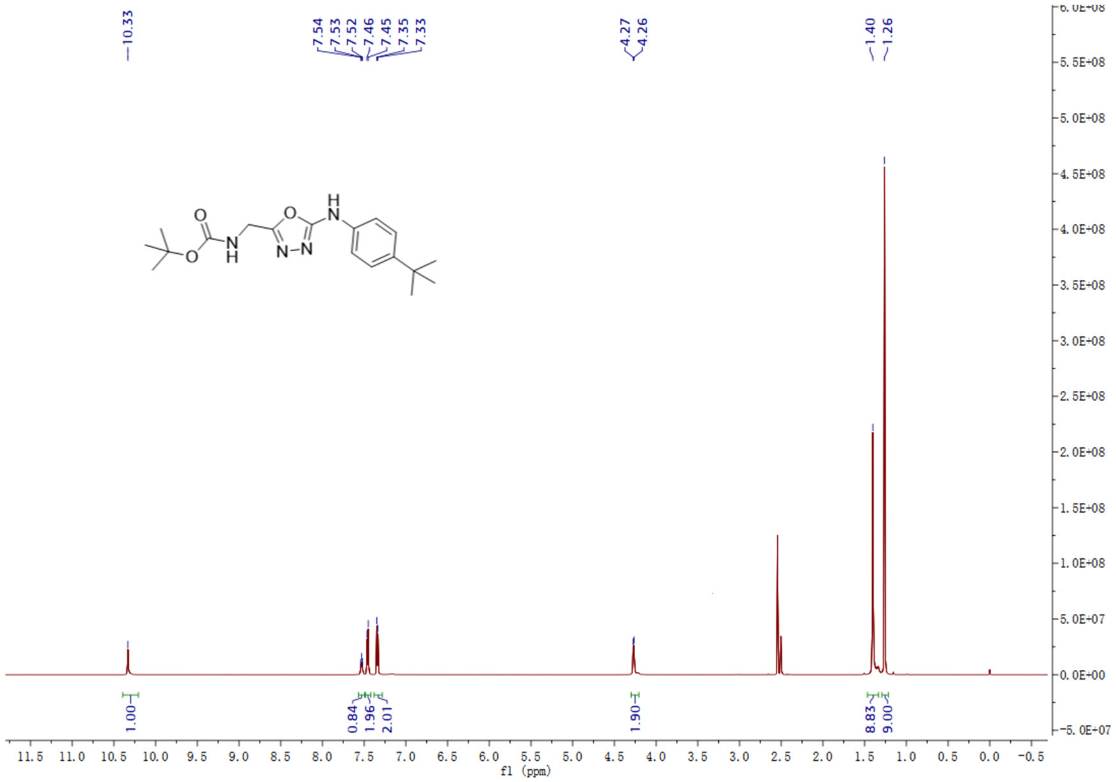

Figure S10. ^{1}H-NMR spectrum of Intermediate **4j**

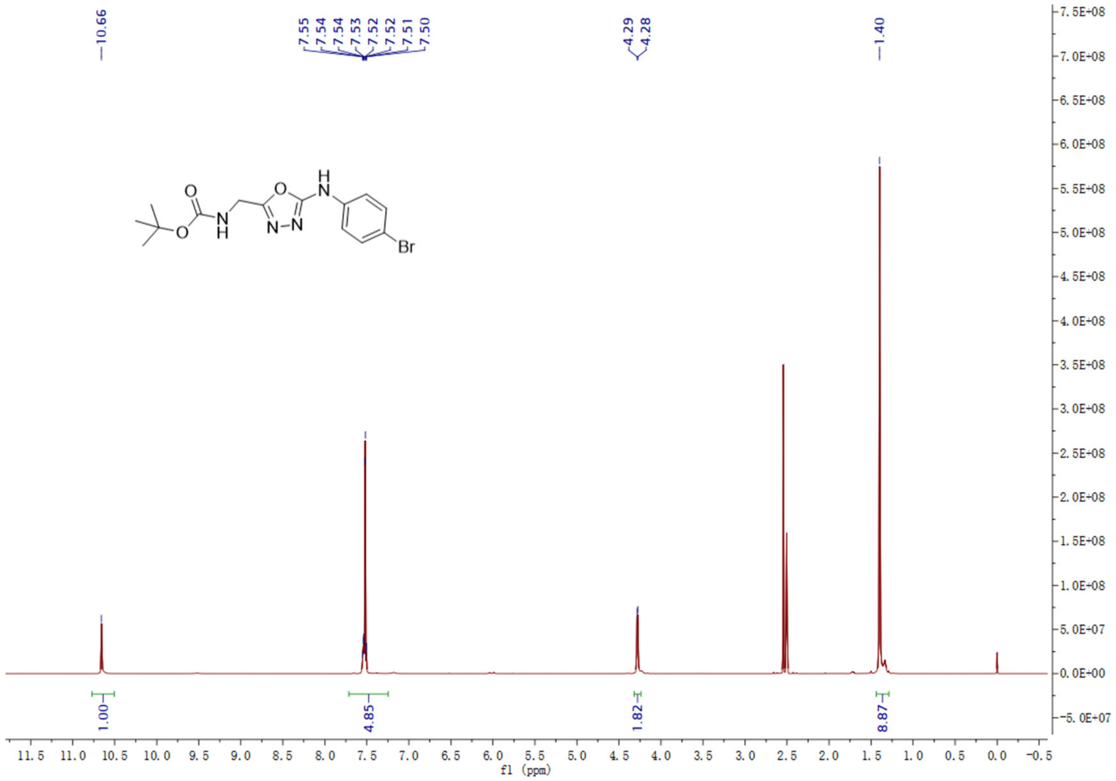

Figure S11. ^{1}H-NMR spectrum of Intermediate **4k**

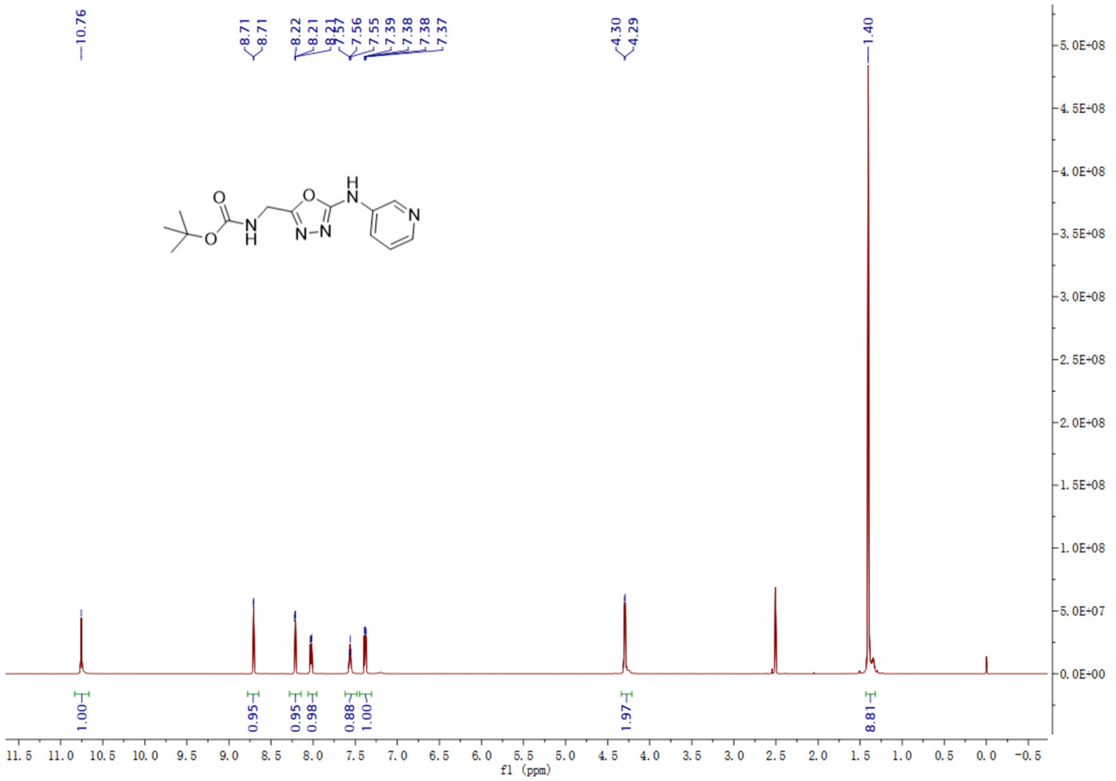

Figure S12. ^{1}H-NMR spectrum of Intermediate **4l**

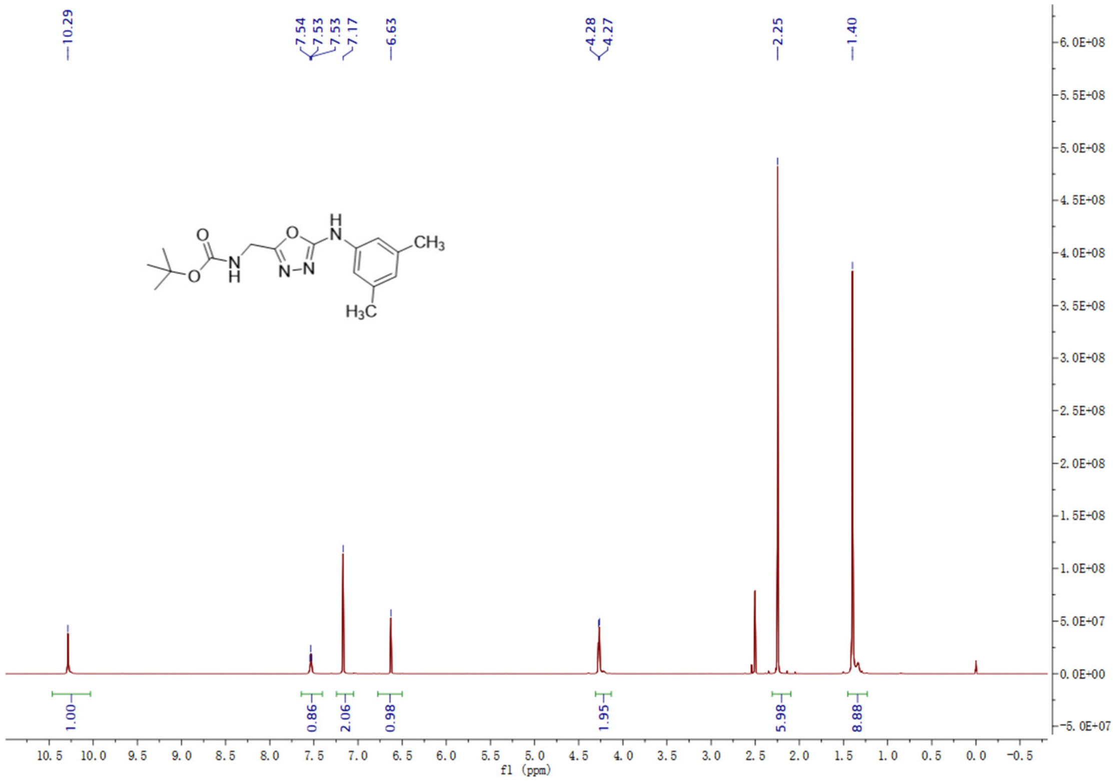

Figure S13. ^{1}H-NMR spectrum of Intermediate **4m**

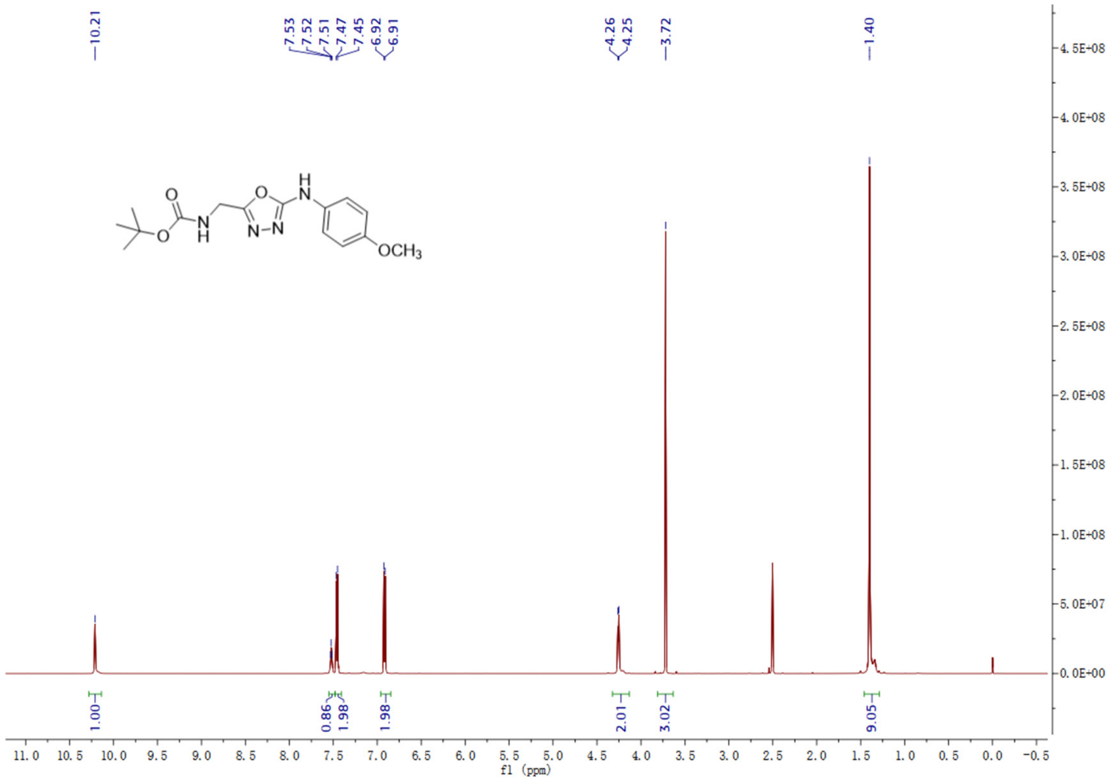

Figure S14. ^{1}H-NMR spectrum of Intermediate **4n**

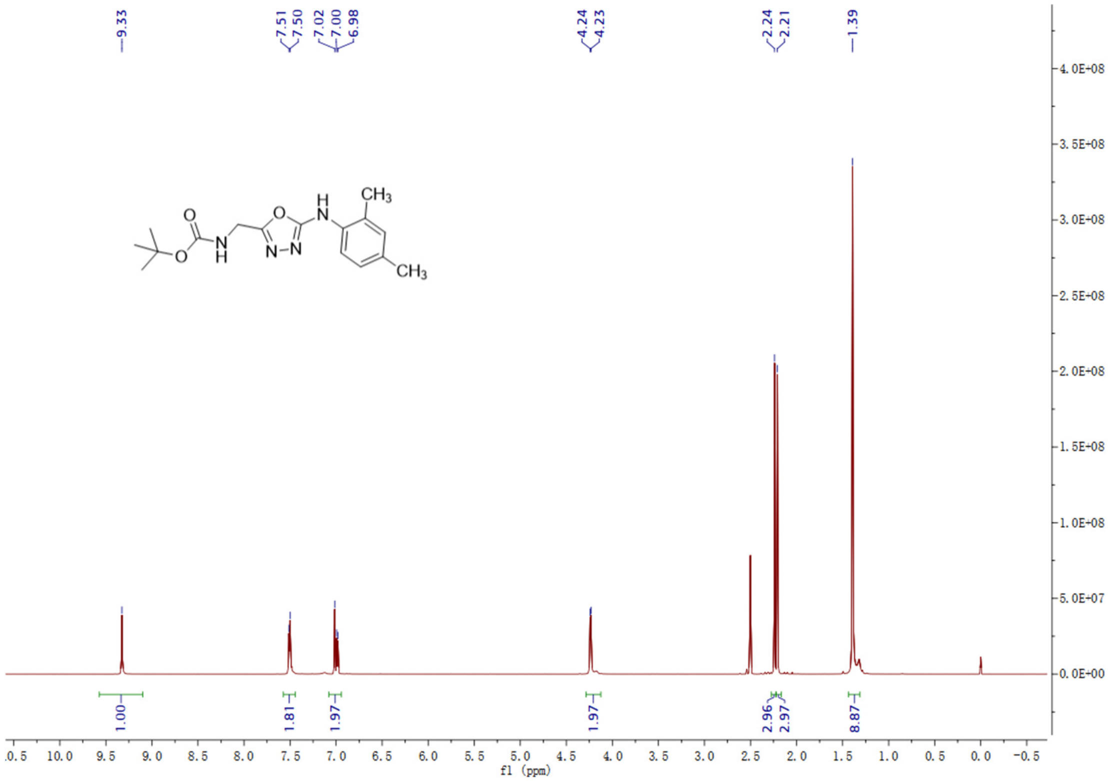

Figure S15. ^{1}H-NMR spectrum of Intermediate **4o**

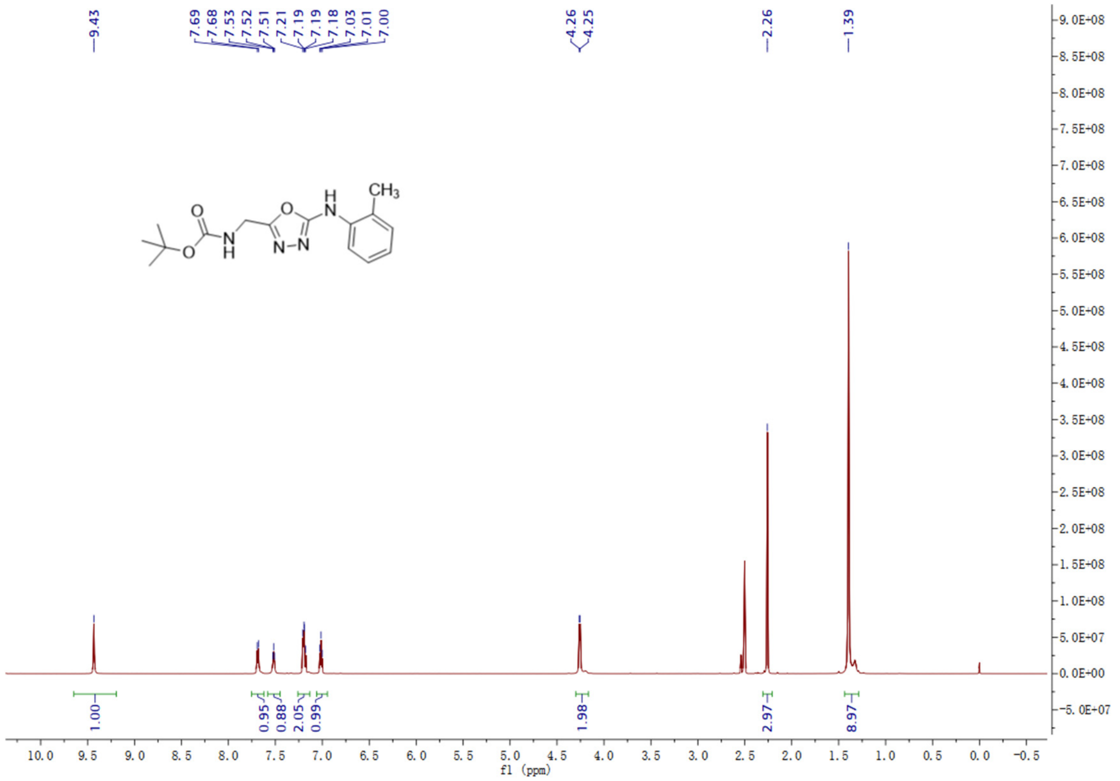

Figure S16. ^{1}H-NMR spectrum of Intermediate **4p**

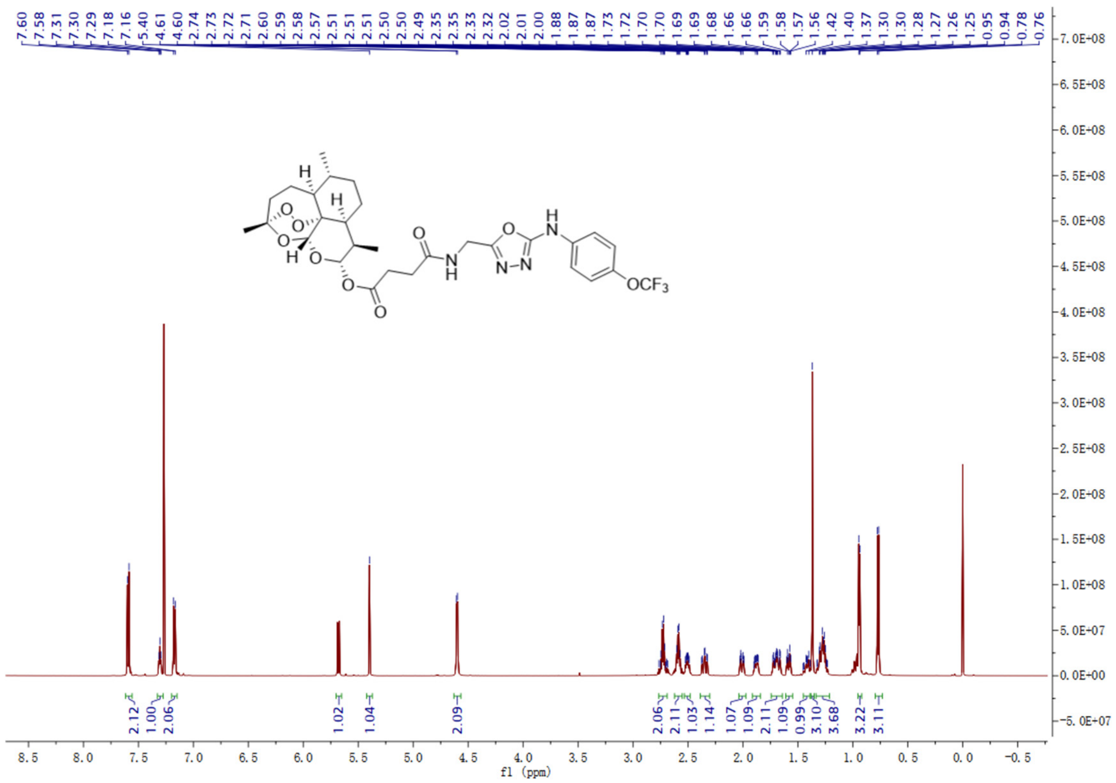

Figure S17. ^{1}H-NMR spectrum of compound **A1**

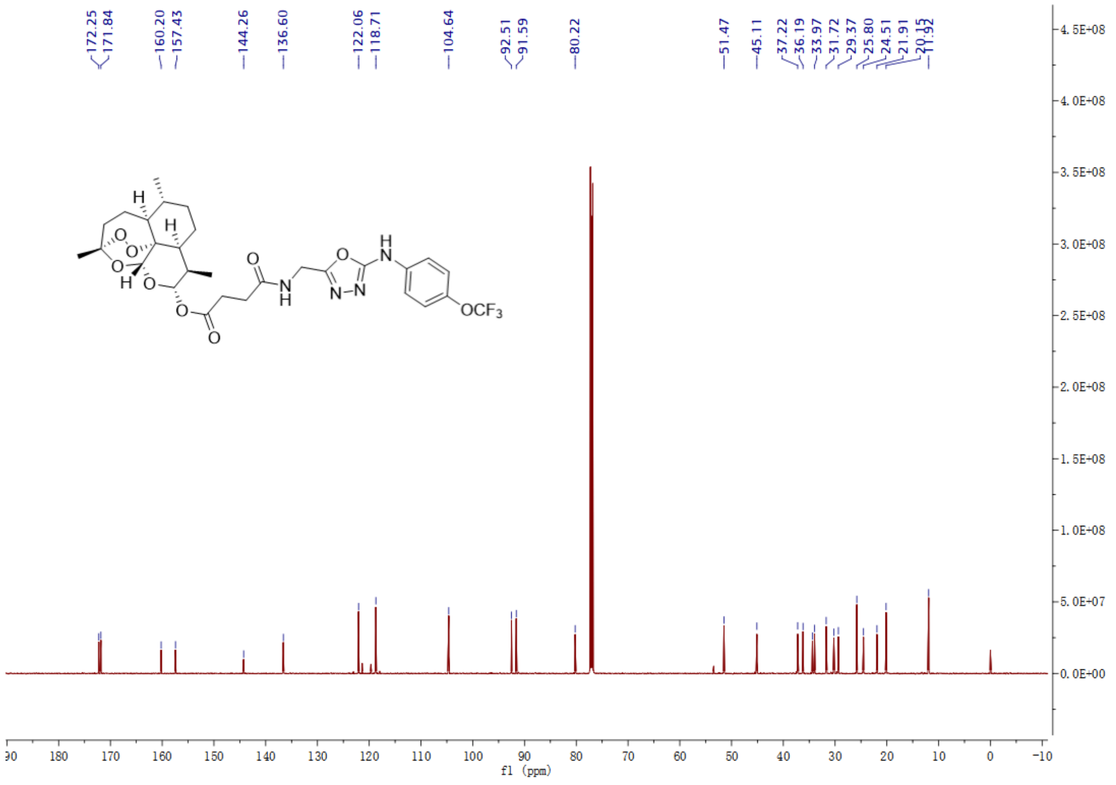

Figure S18. ^{13}C-NMR spectrum of compound **A1**

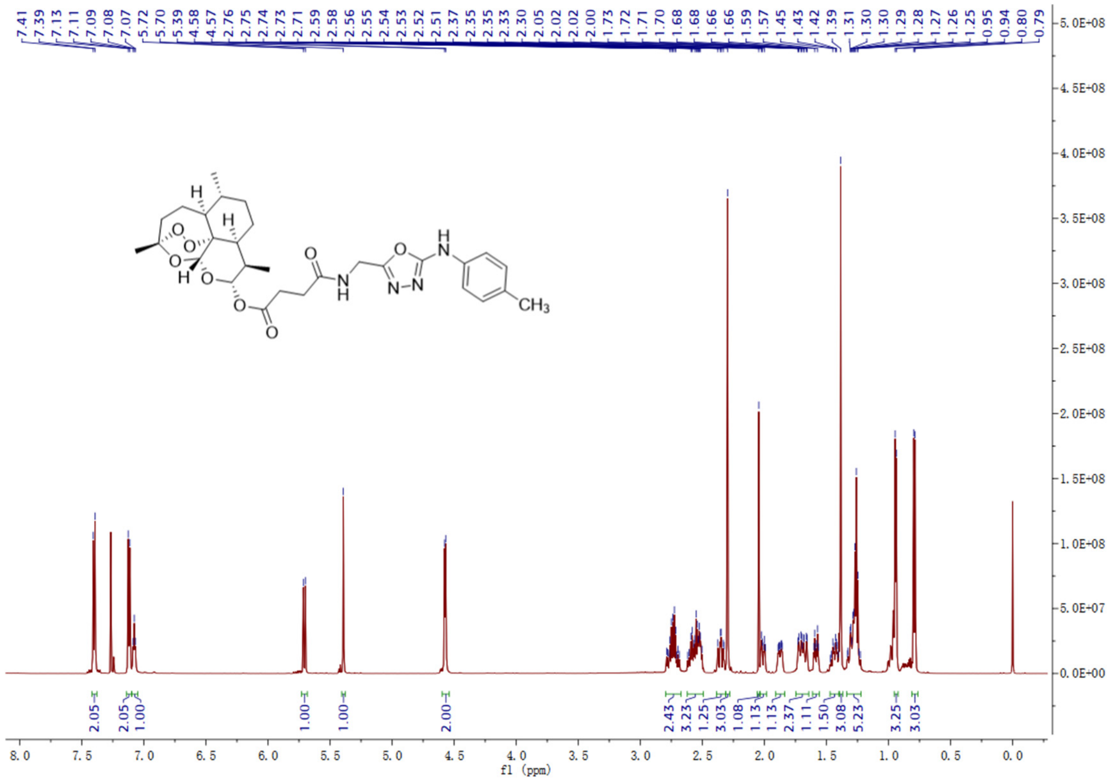

Figure S19. ^{1}H-NMR spectrum of compound **A2**

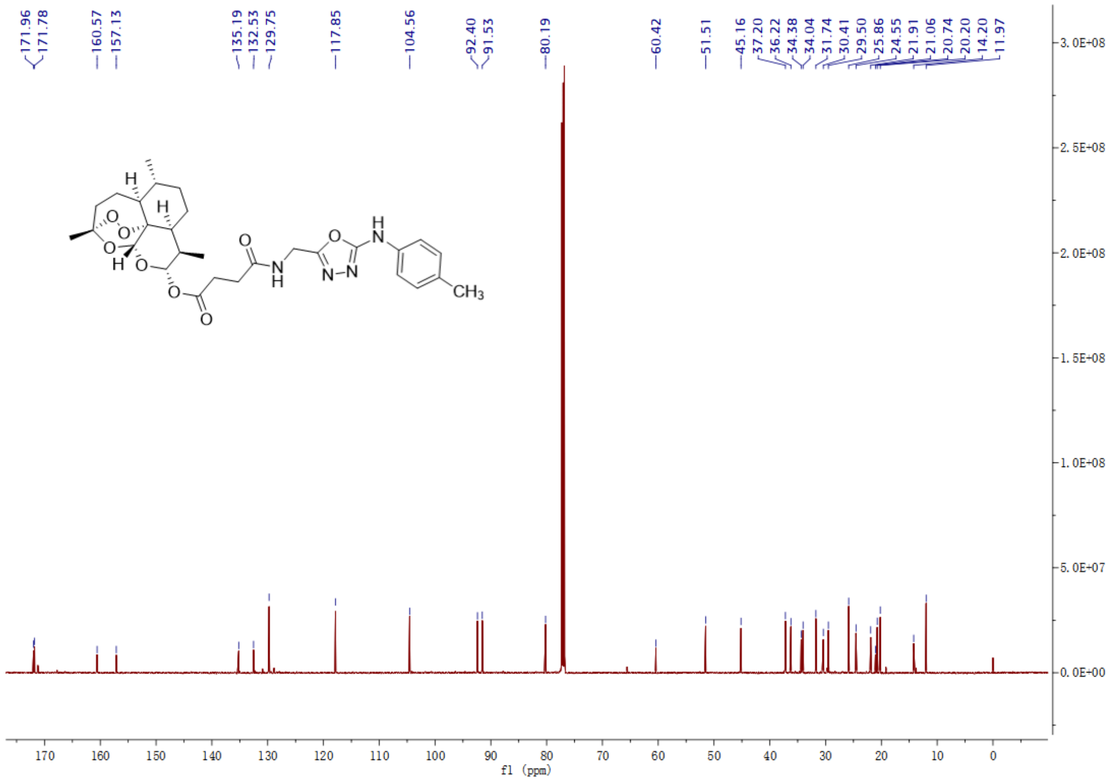

Figure S20. ^{13}C-NMR spectrum of compound **A2**

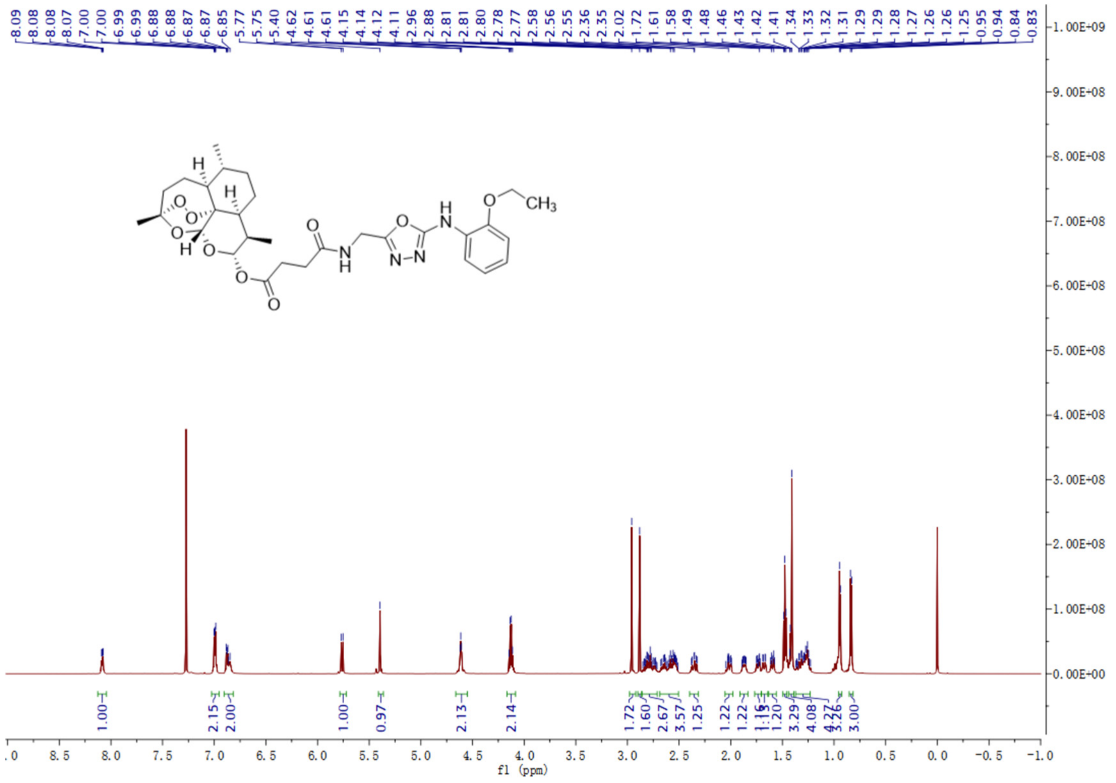

Figure S21. ^{1}H-NMR spectrum of compound **A3**

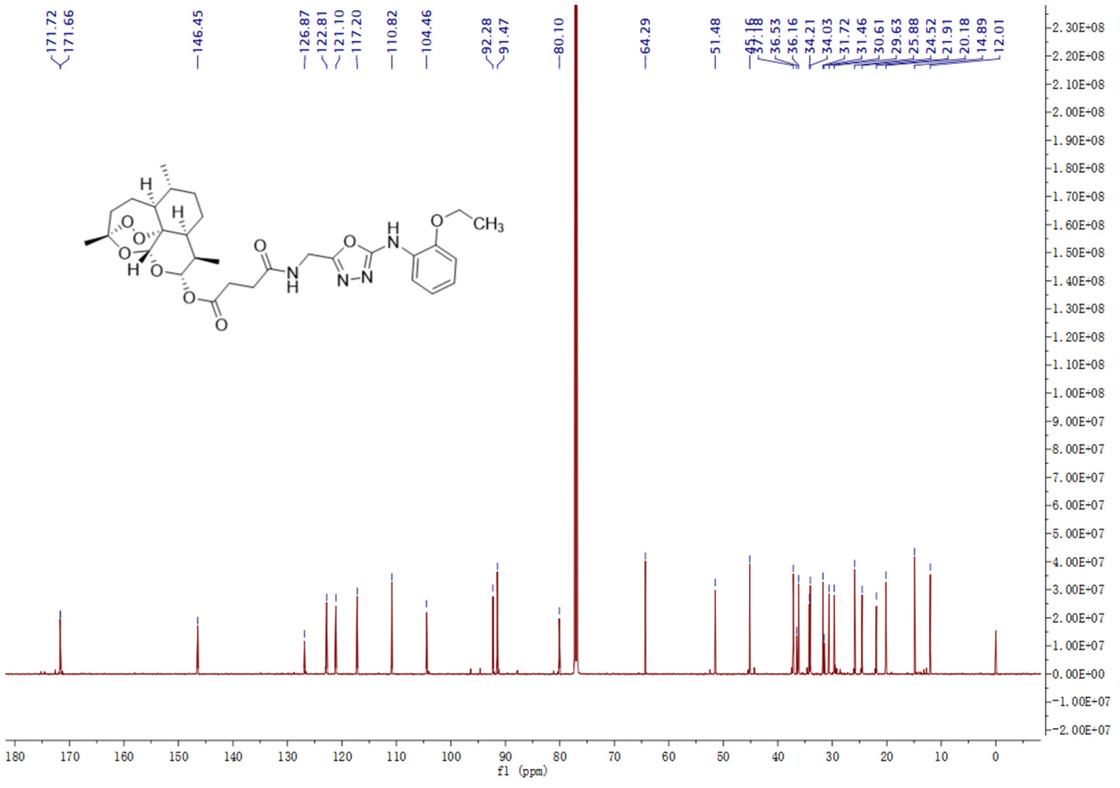

Figure S22. ^{13}C-NMR spectrum of compound **A3**

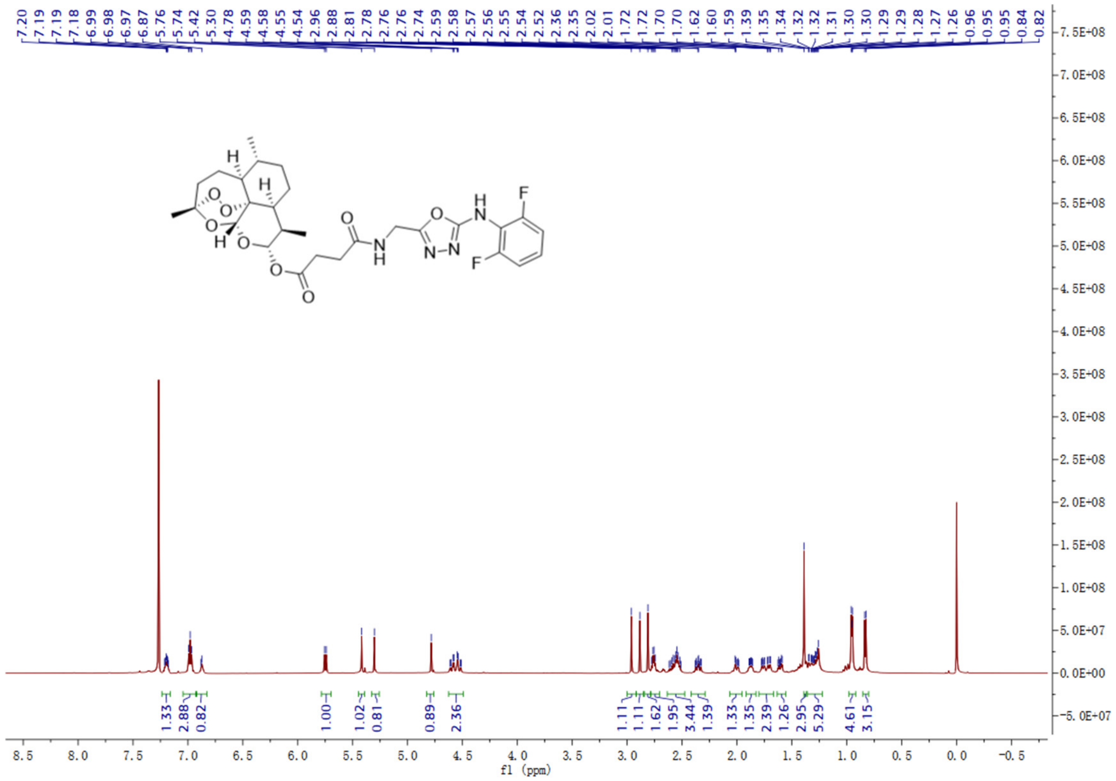

Figure S23. ^{1}H-NMR spectrum of compound **A4**

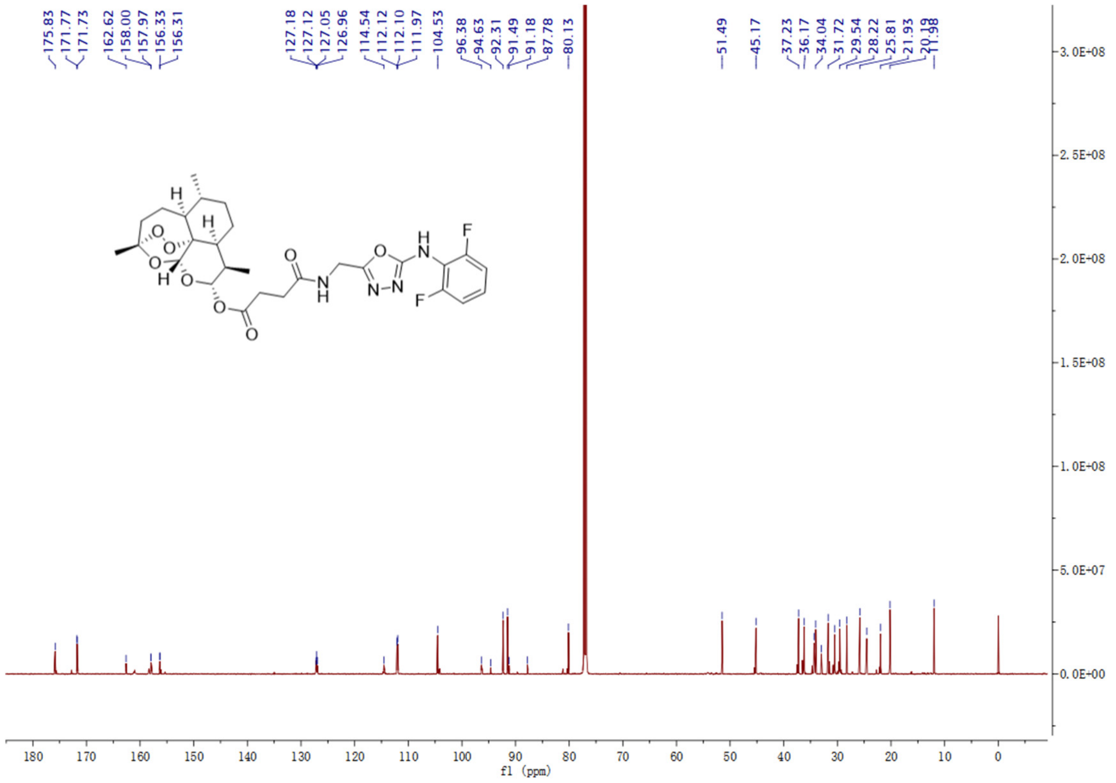

Figure S24. ^{13}C-NMR spectrum of compound **A4**

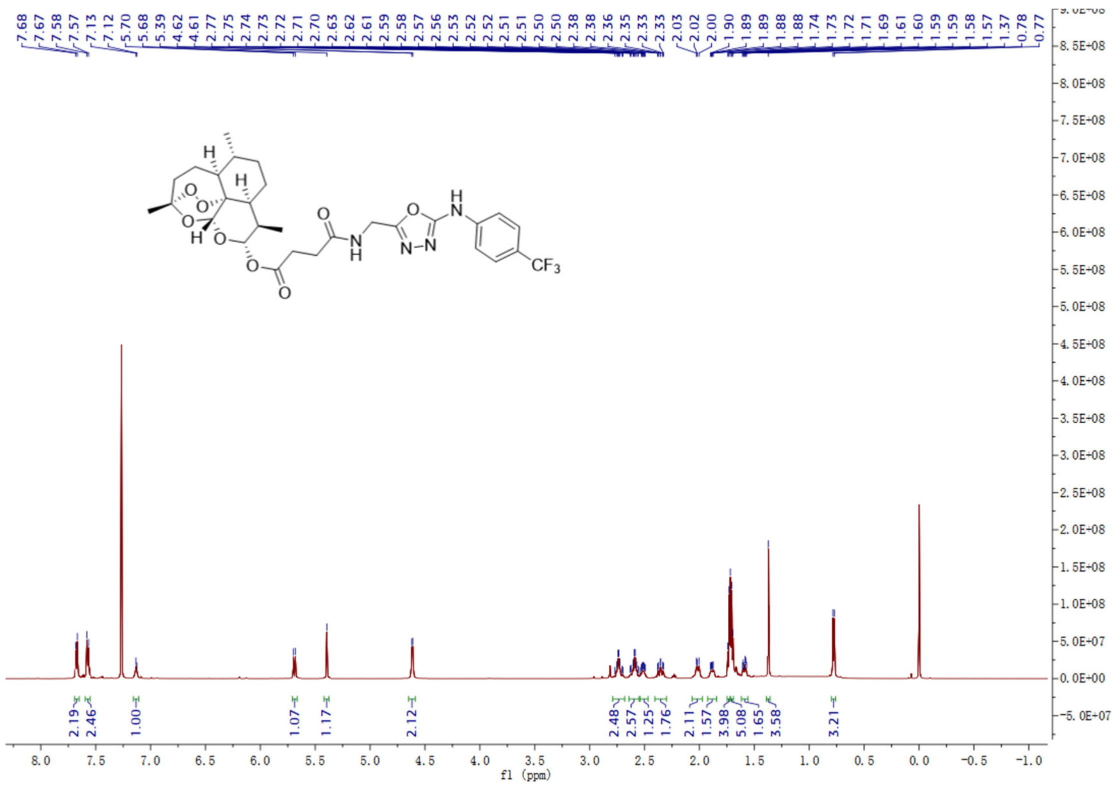

Figure S25. ^{1}H-NMR spectrum of compound **A5**

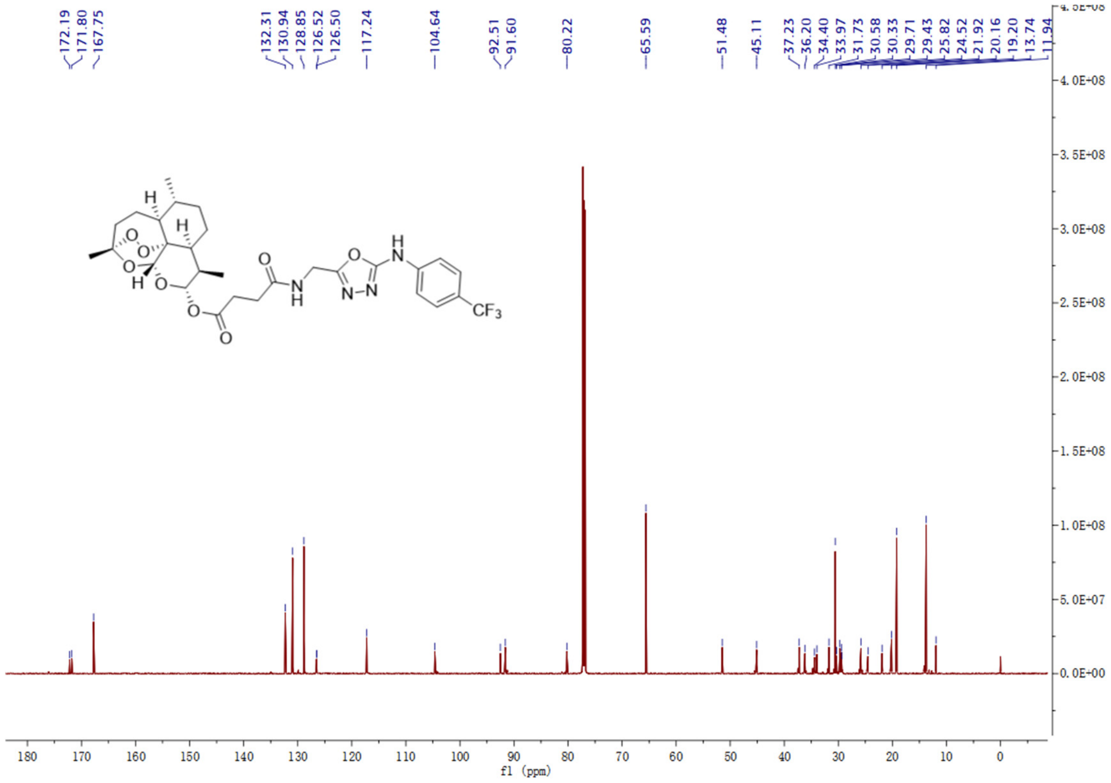

Figure S26. ^{13}C-NMR spectrum of compound **A5**

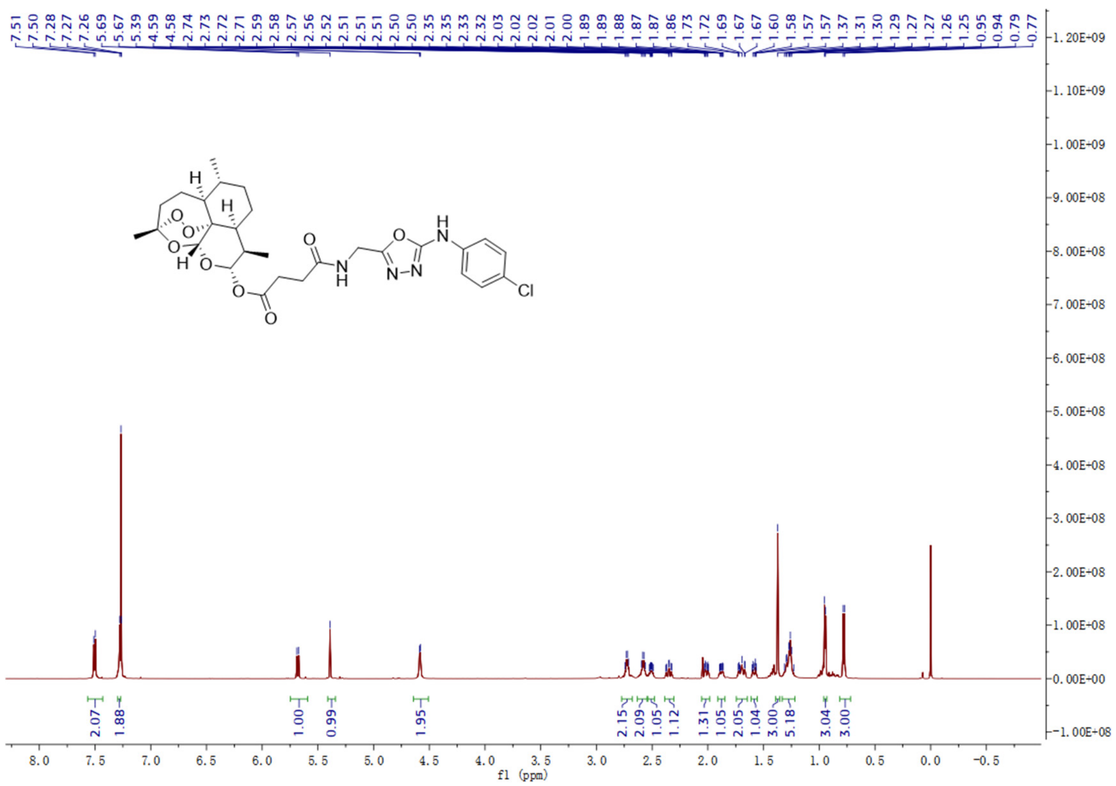

Figure S27. ^{1}H-NMR spectrum of compound **A6**

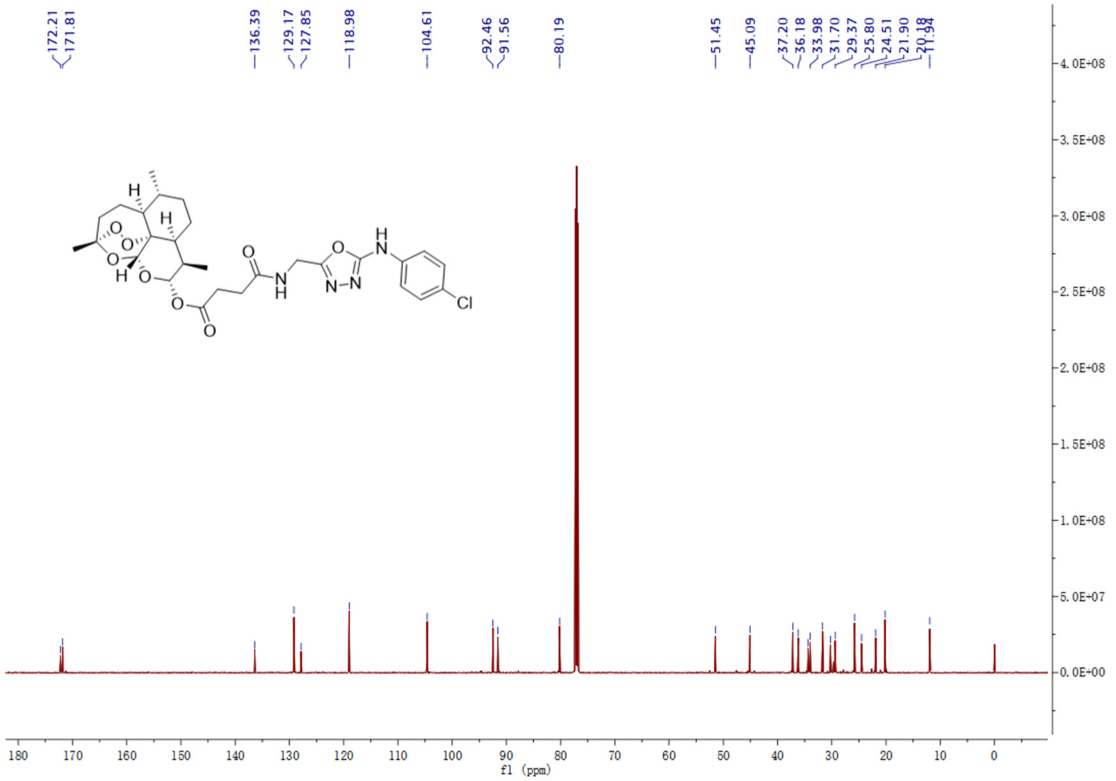

Figure S28. ^{13}C-NMR spectrum of compound **A6**

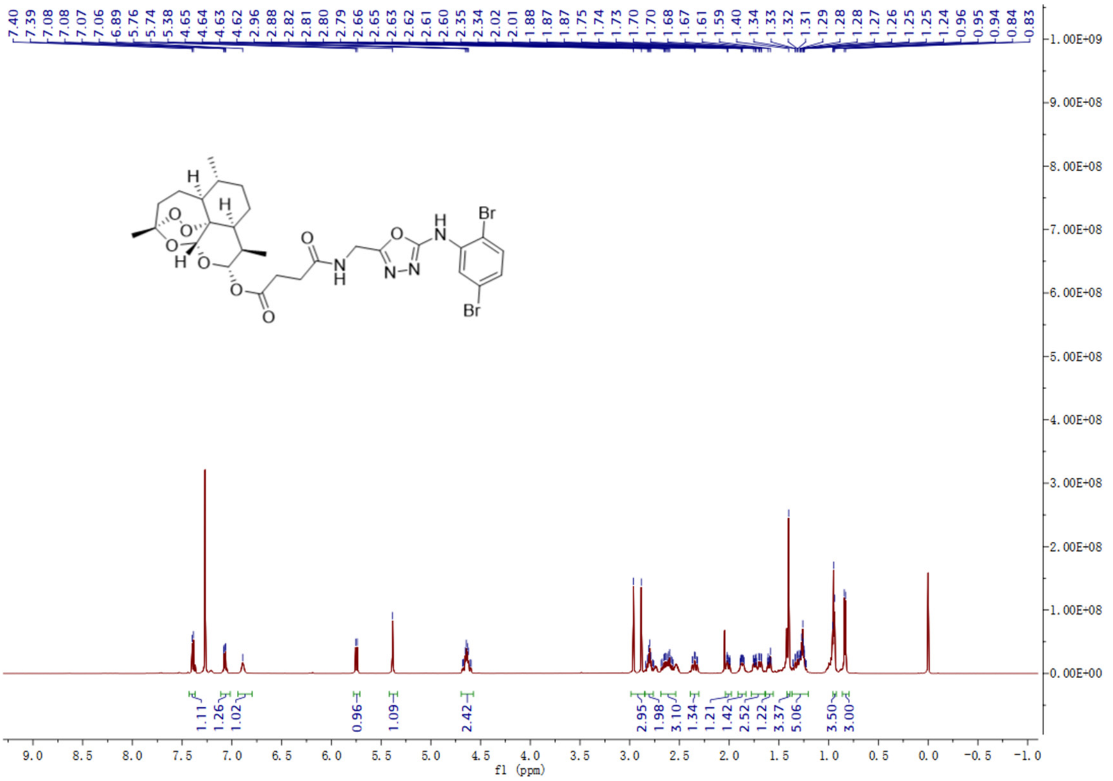

Figure S29. ^{1}H-NMR spectrum of compound **A7**

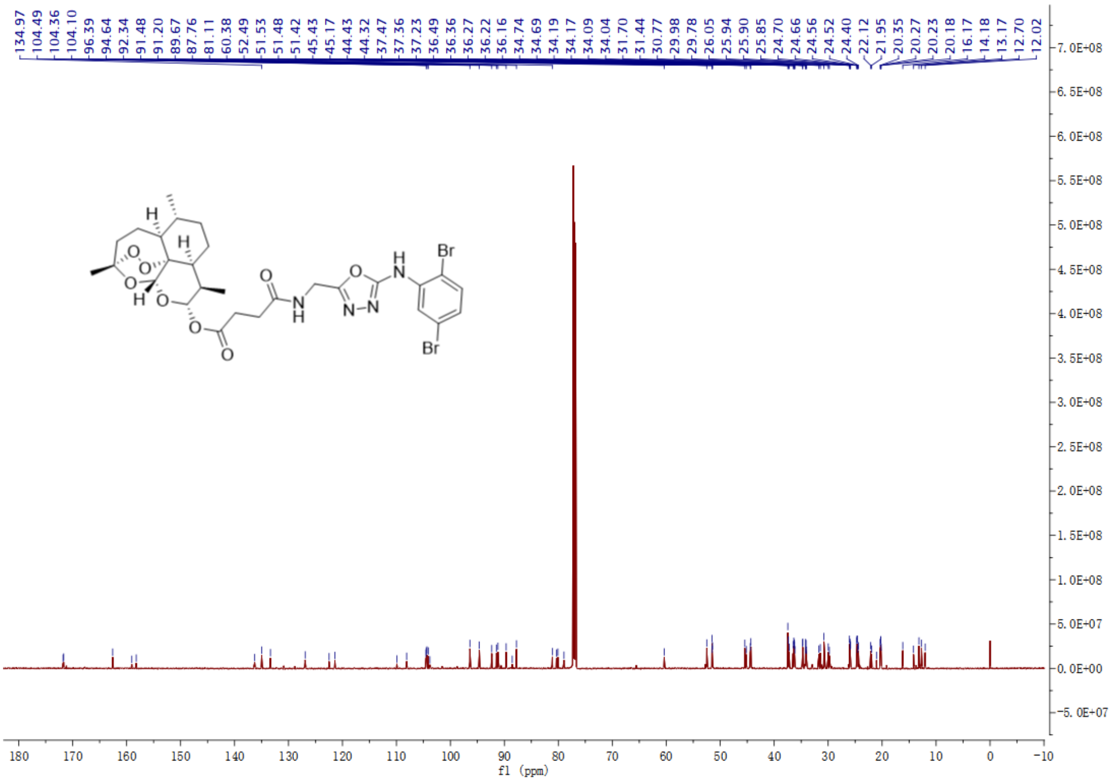

Figure S30. ^{13}C-NMR spectrum of compound **A7**

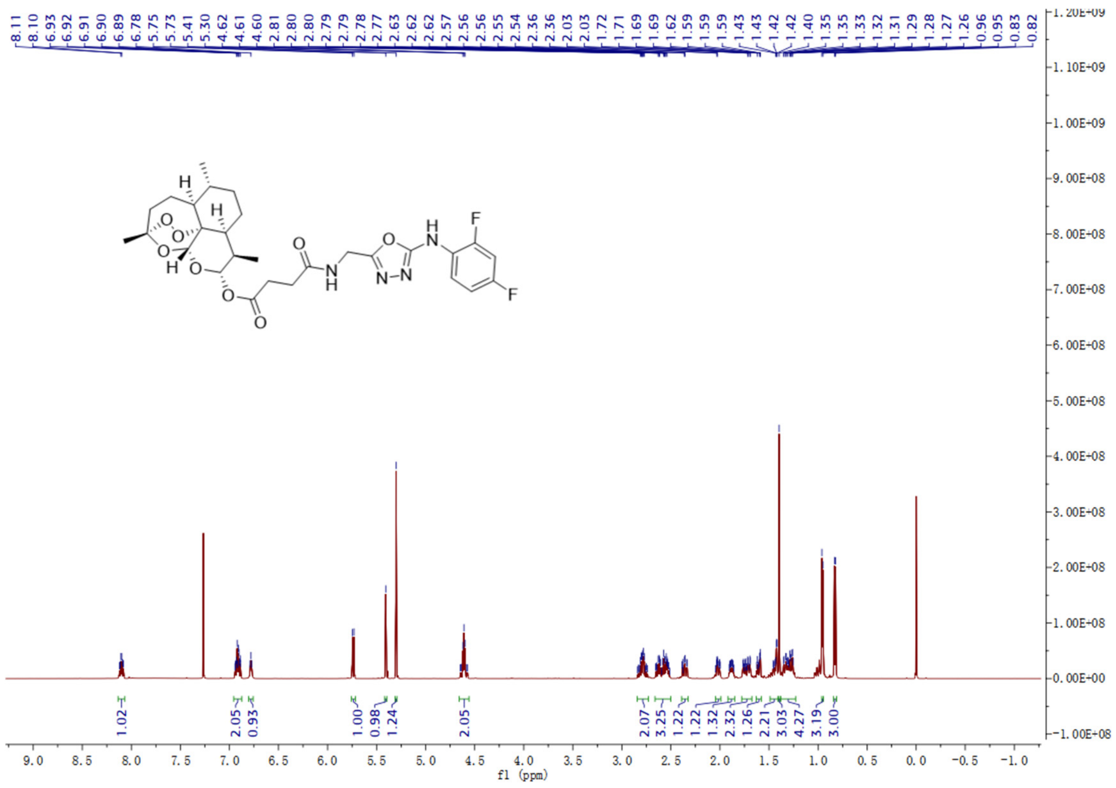

Figure S31. ^{1}H-NMR spectrum of compound **A8**

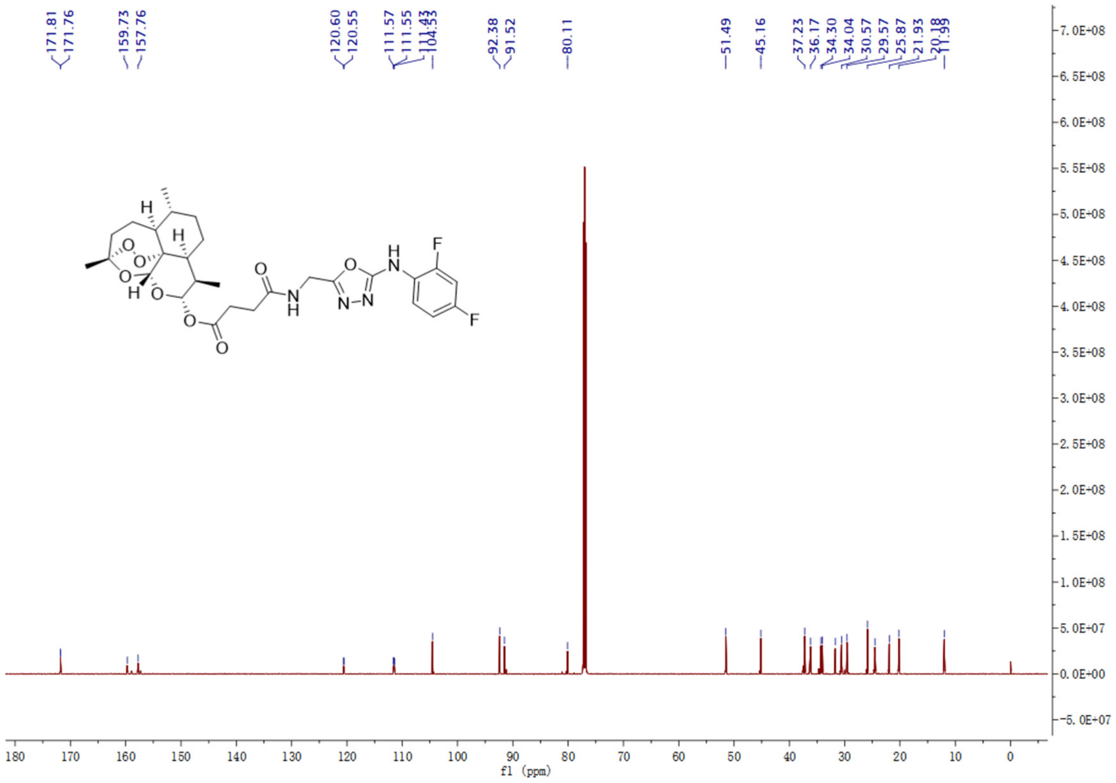

Figure S32. ^{13}C-NMR spectrum of compound **A8**

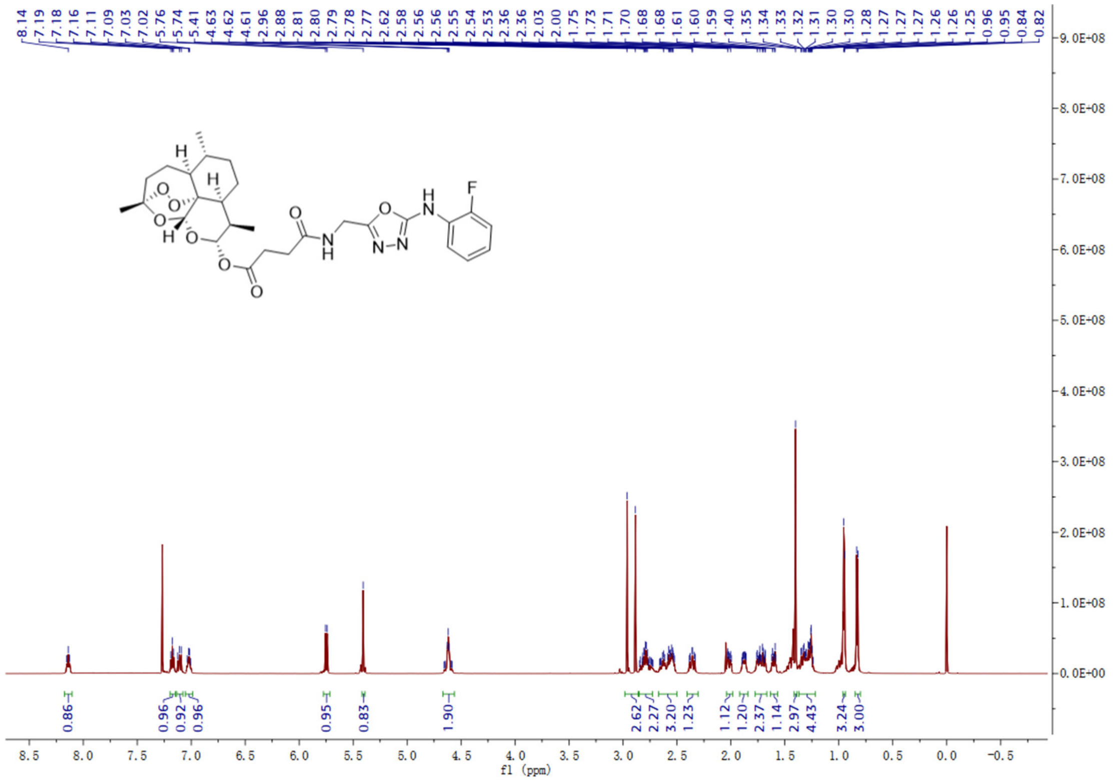

Figure S33. ^{1}H-NMR spectrum of compound **A9**

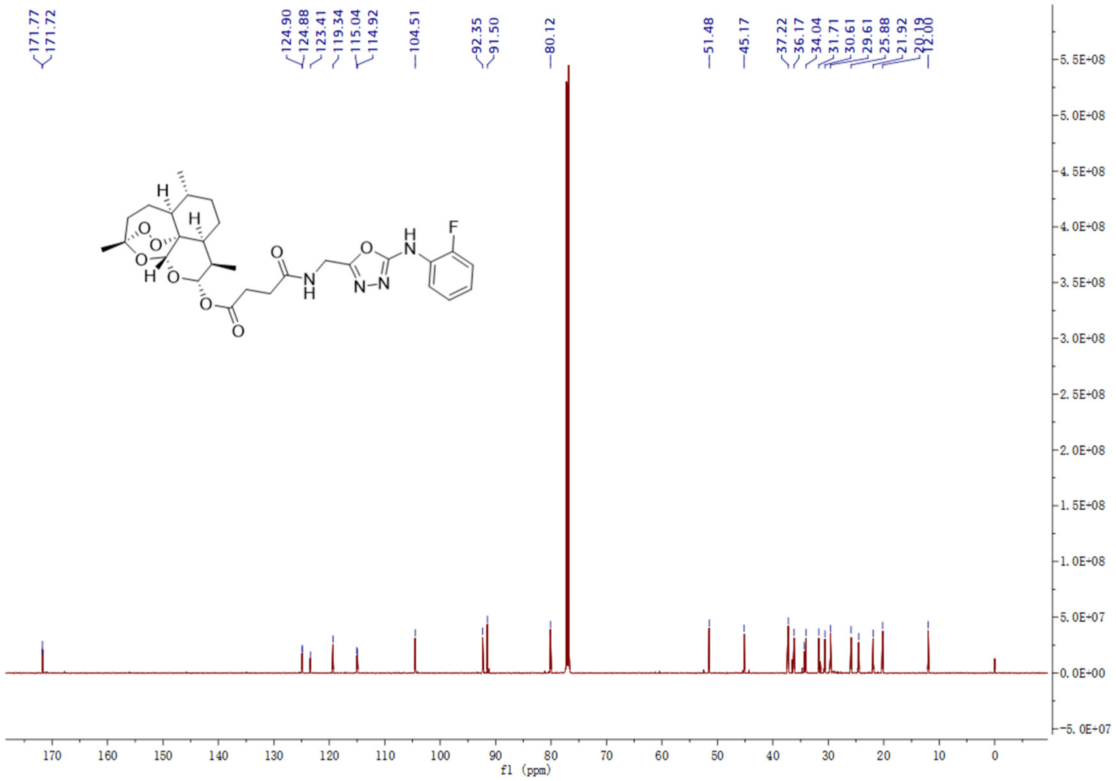

Figure S34. ^{13}C-NMR spectrum of compound **A9**

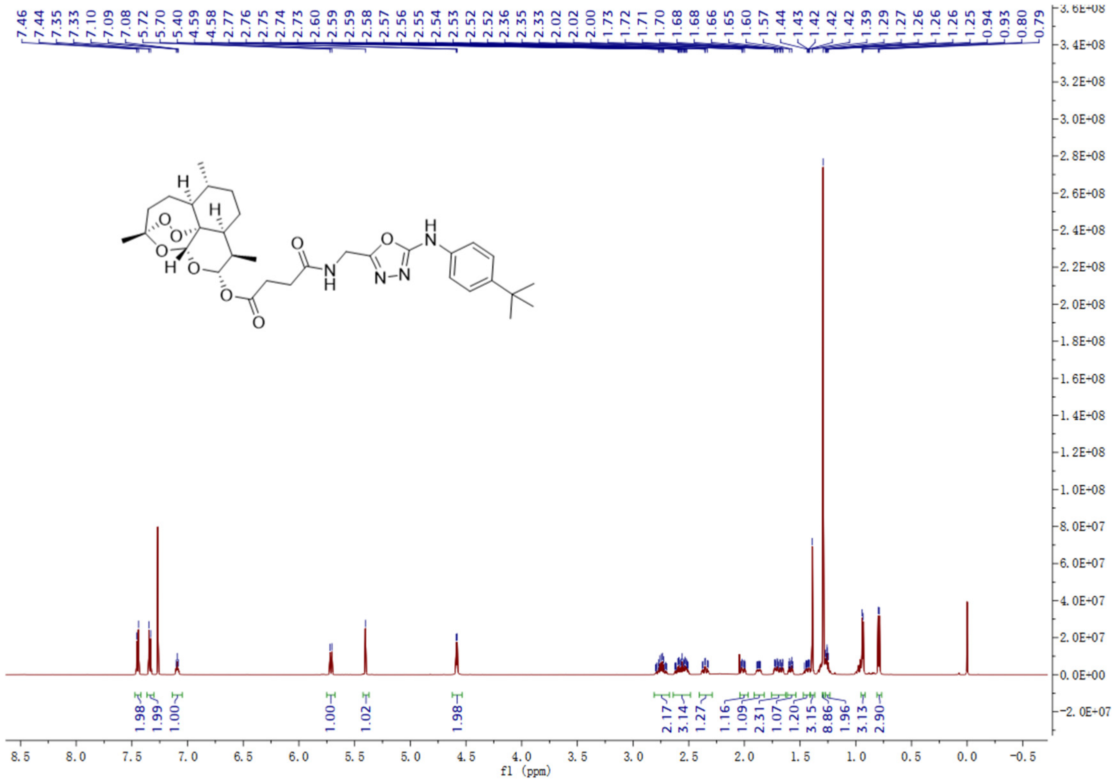

Figure S35. ^{1}H-NMR spectrum of compound **A10**

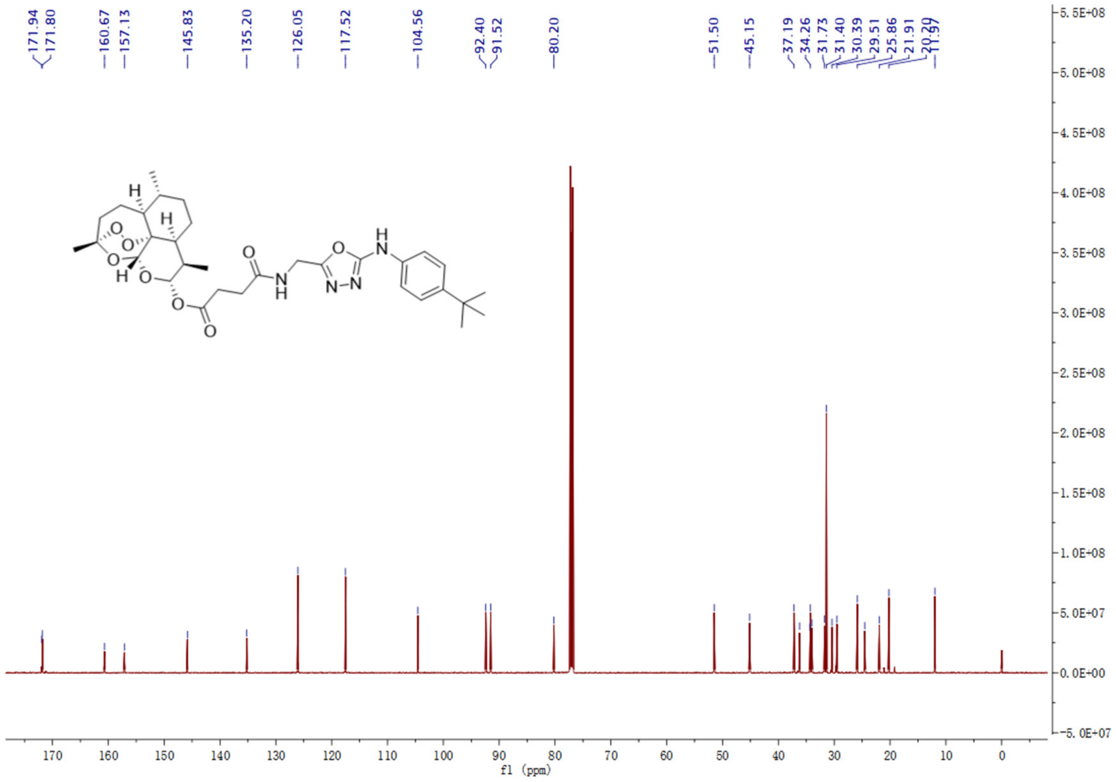

Figure S36. ^{13}C-NMR spectrum of compound **A10**

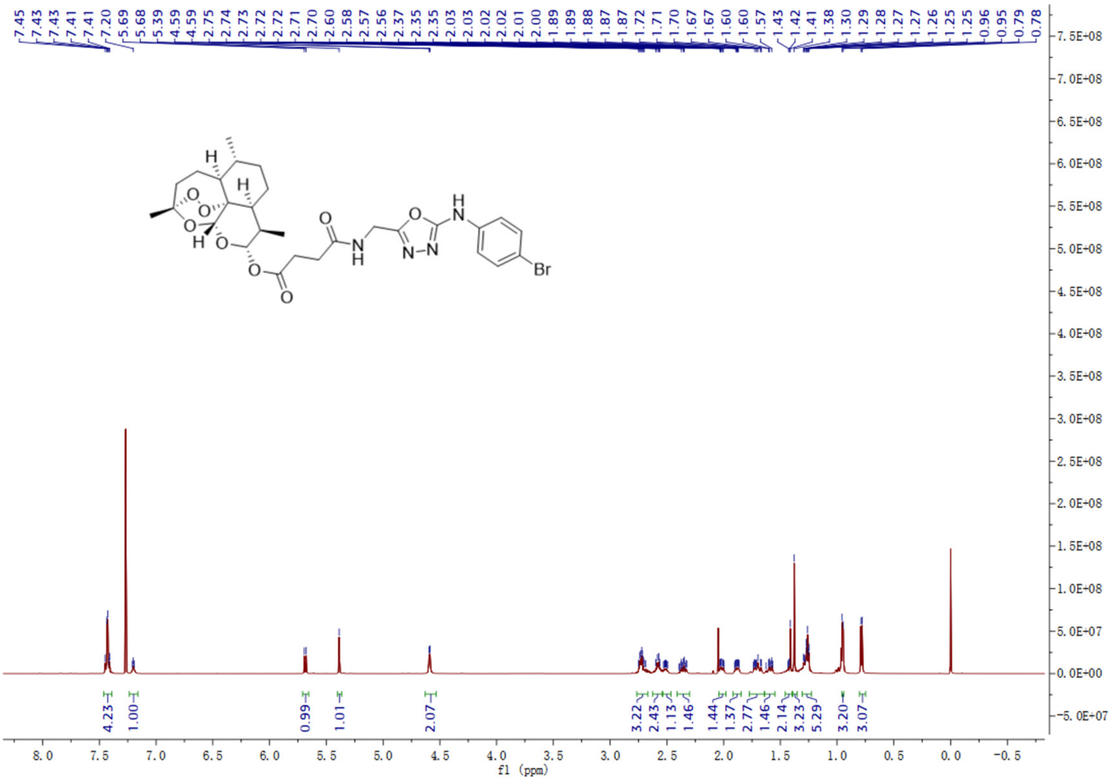

Figure S37. ^{1}H-NMR spectrum of compound **A11**

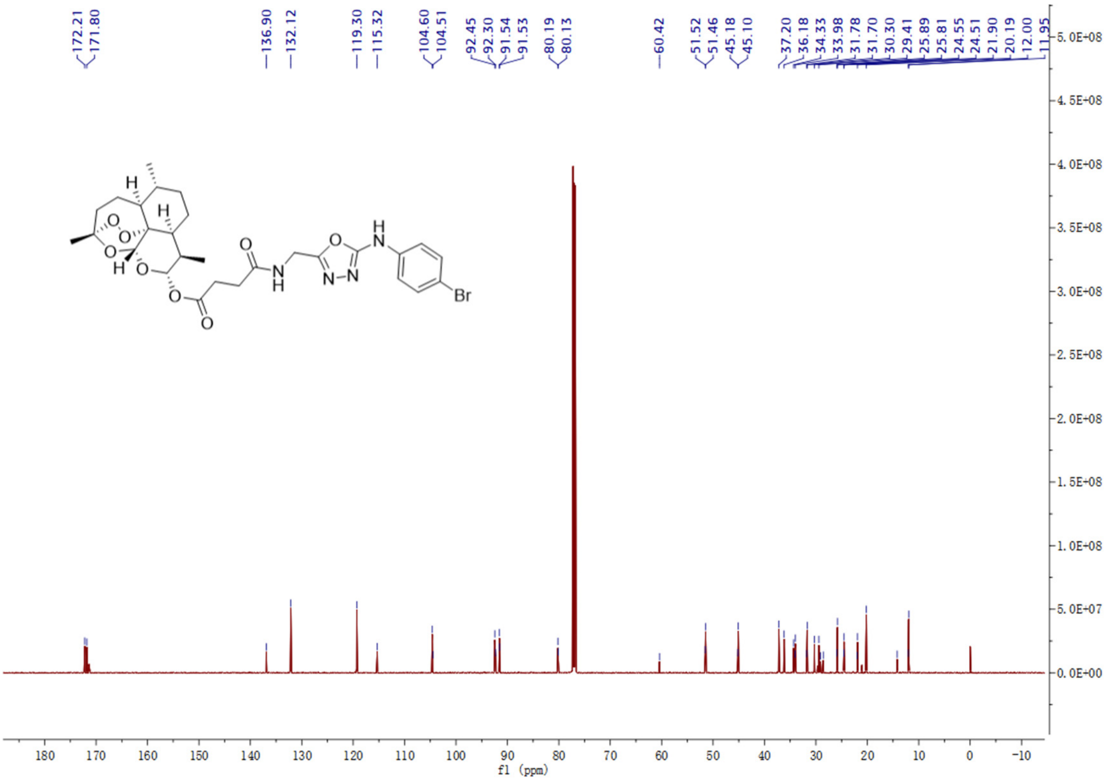

Figure S38. ^{13}C-NMR spectrum of compound **A11**

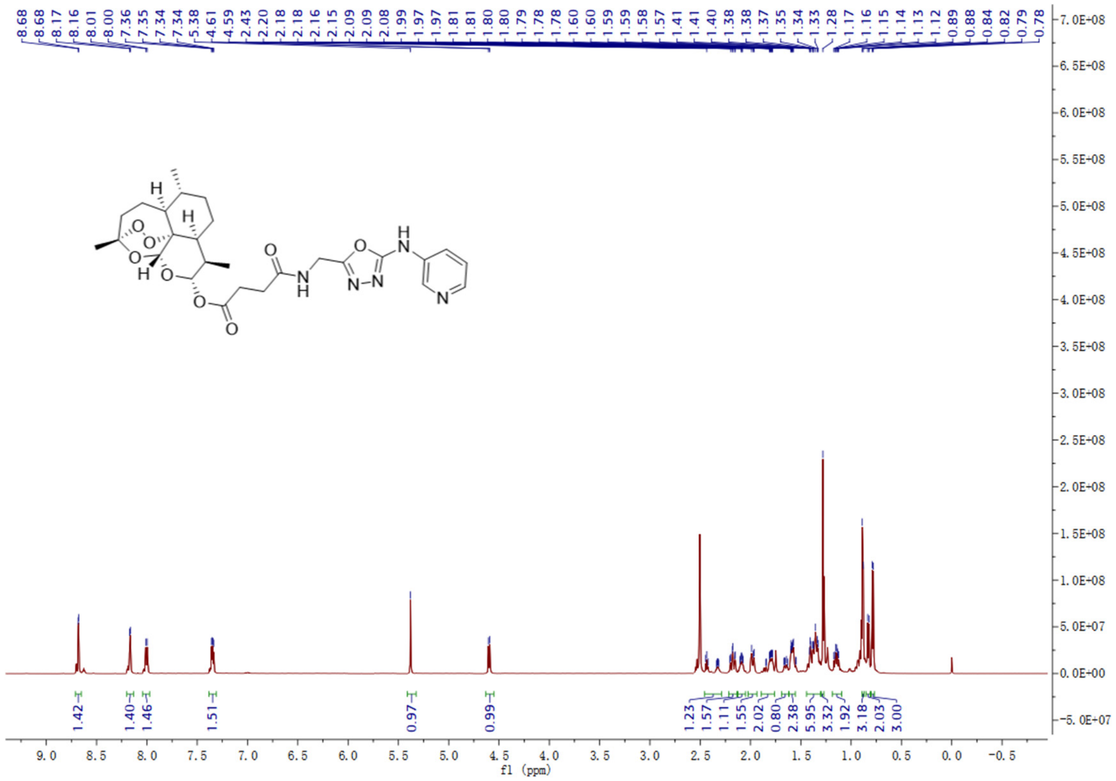

Figure S39. ^{1}H-NMR spectrum of compound **A12**

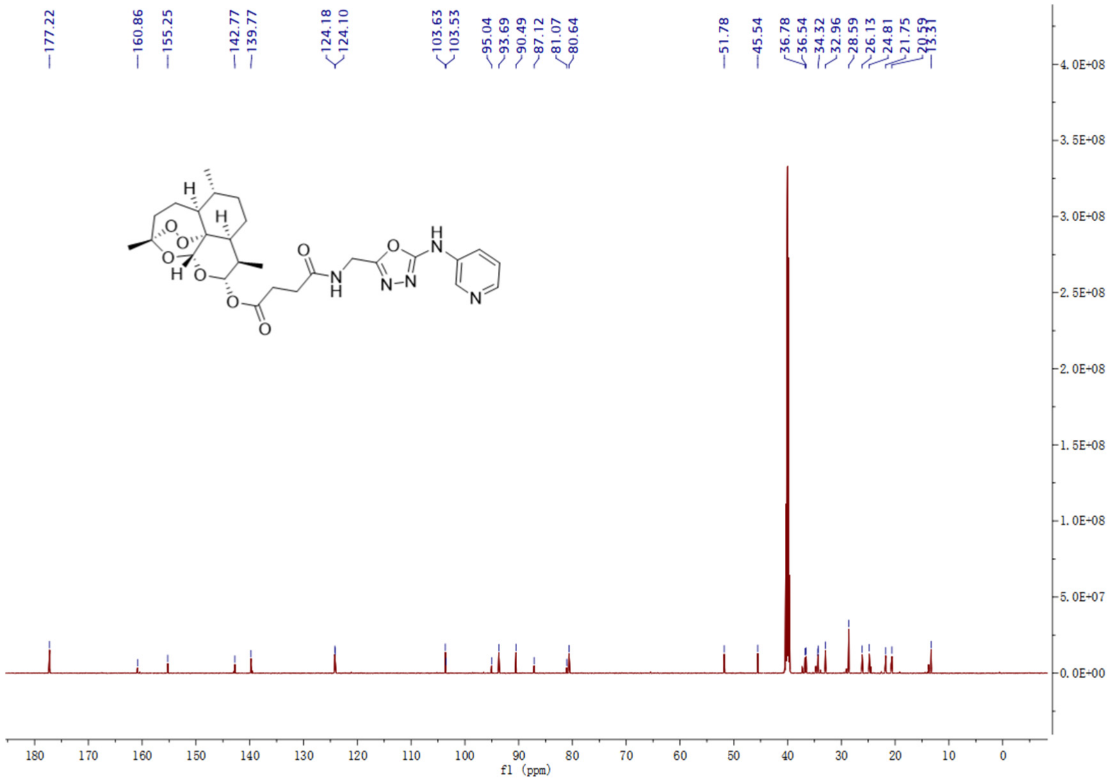

Figure S40. ^{13}C-NMR spectrum of compound **A12**

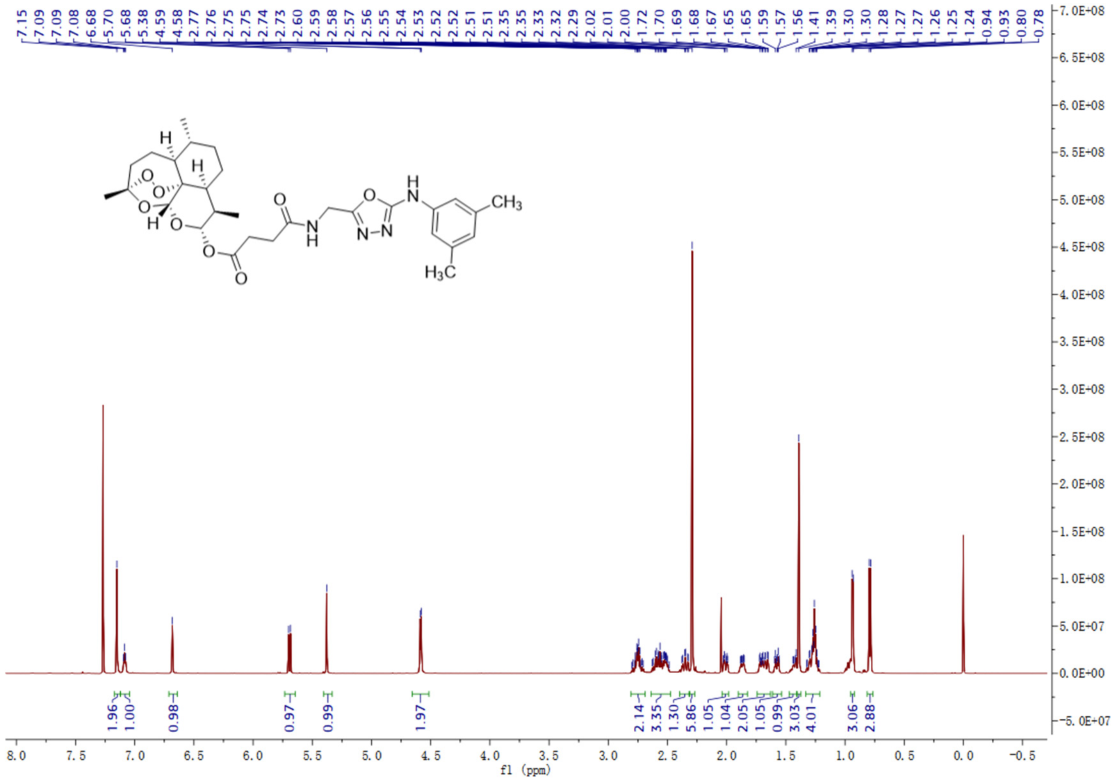

Figure S41. ^{1}H-NMR spectrum of compound **A13**

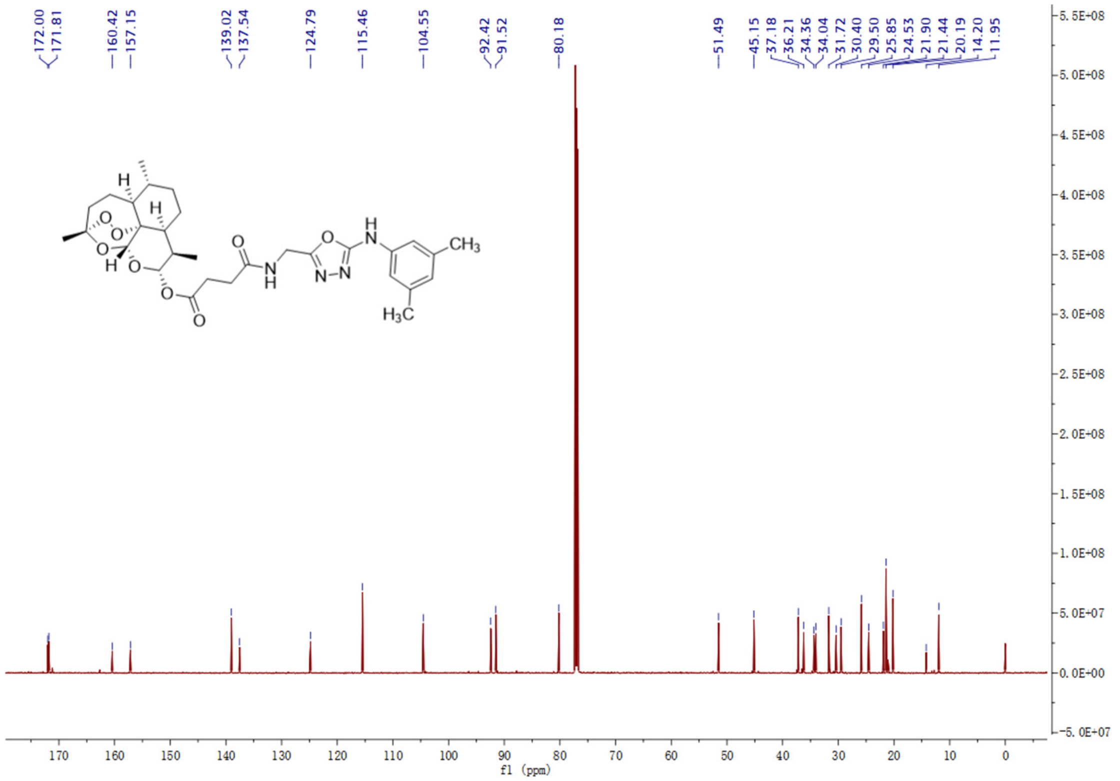

Figure S42. ^{13}C-NMR spectrum of compound **A13**

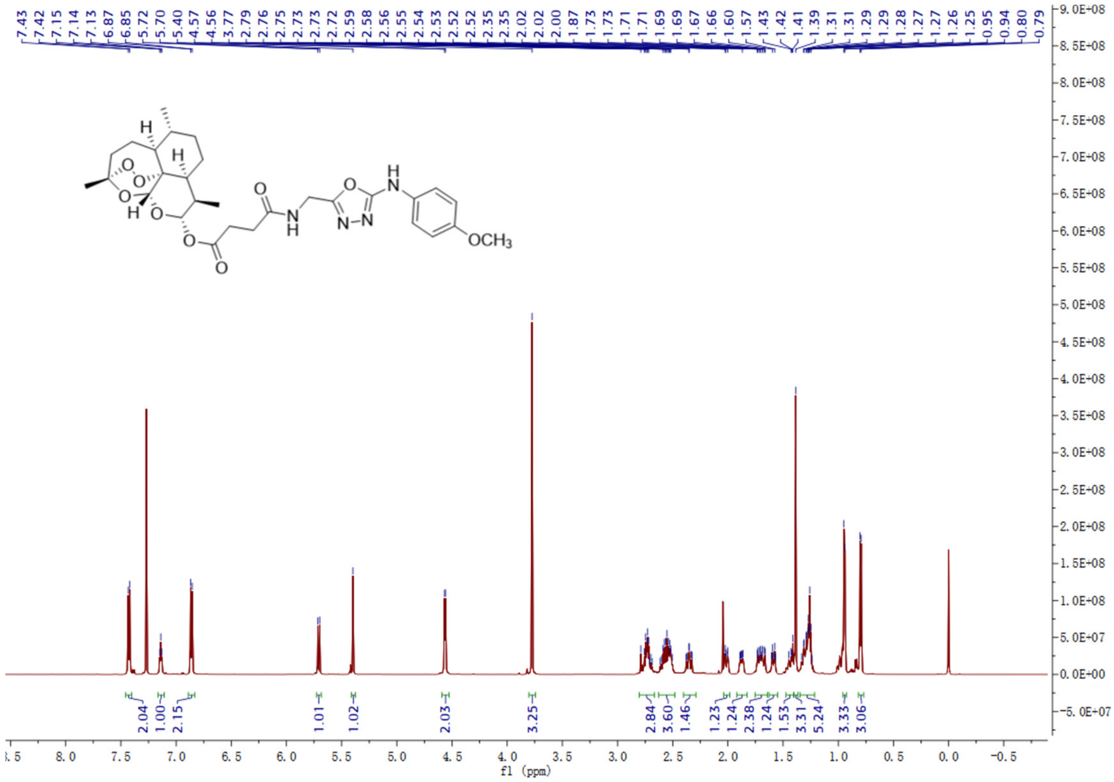

Figure S43. ^{1}H-NMR spectrum of compound **A14**

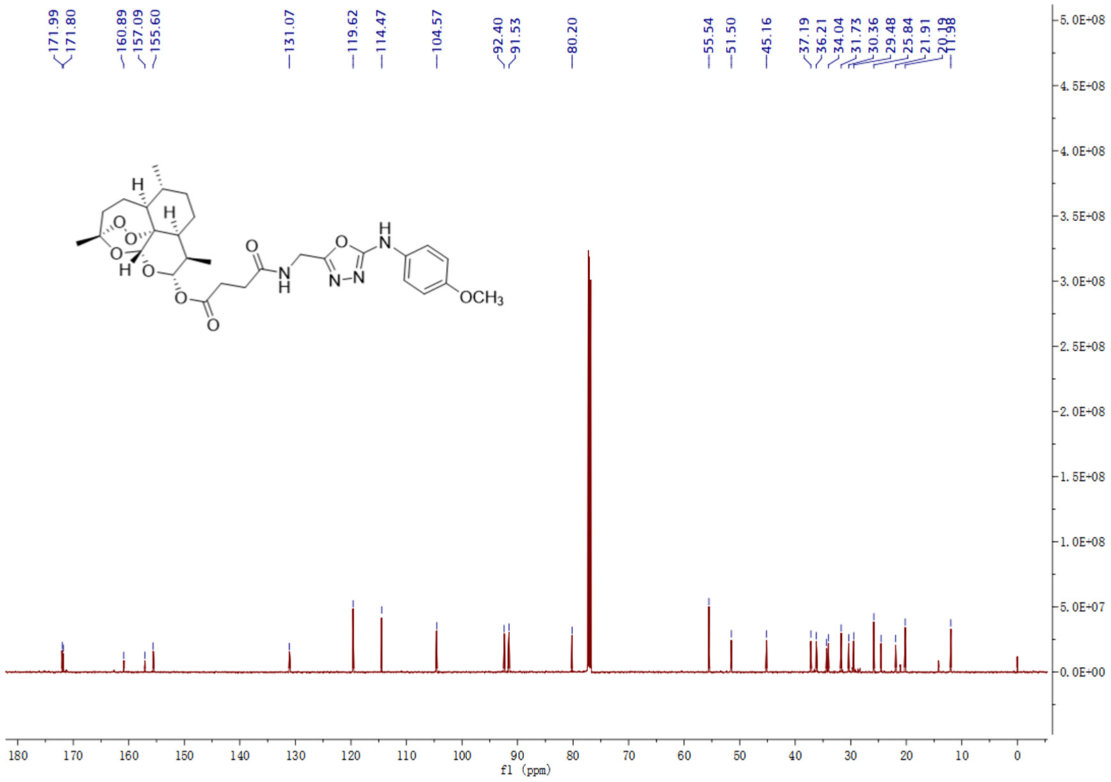

Figure S44. ^{13}C-NMR spectrum of compound **A14**

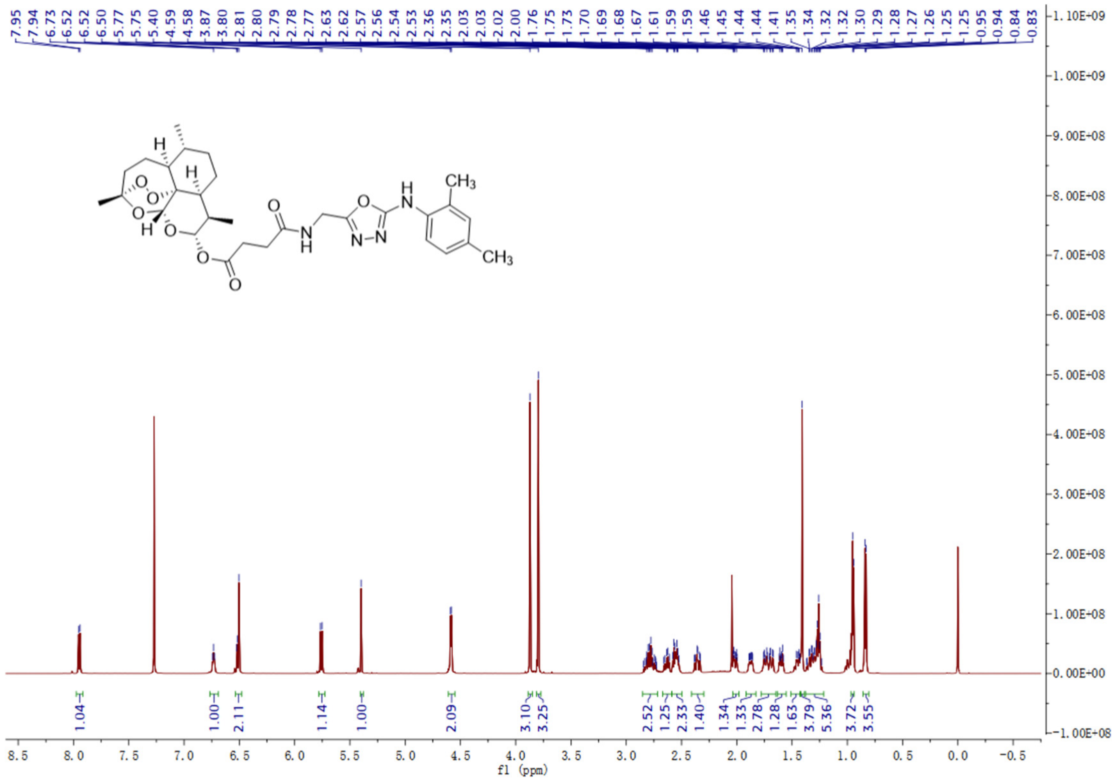

Figure S45. ^{1}H-NMR spectrum of compound **A15**

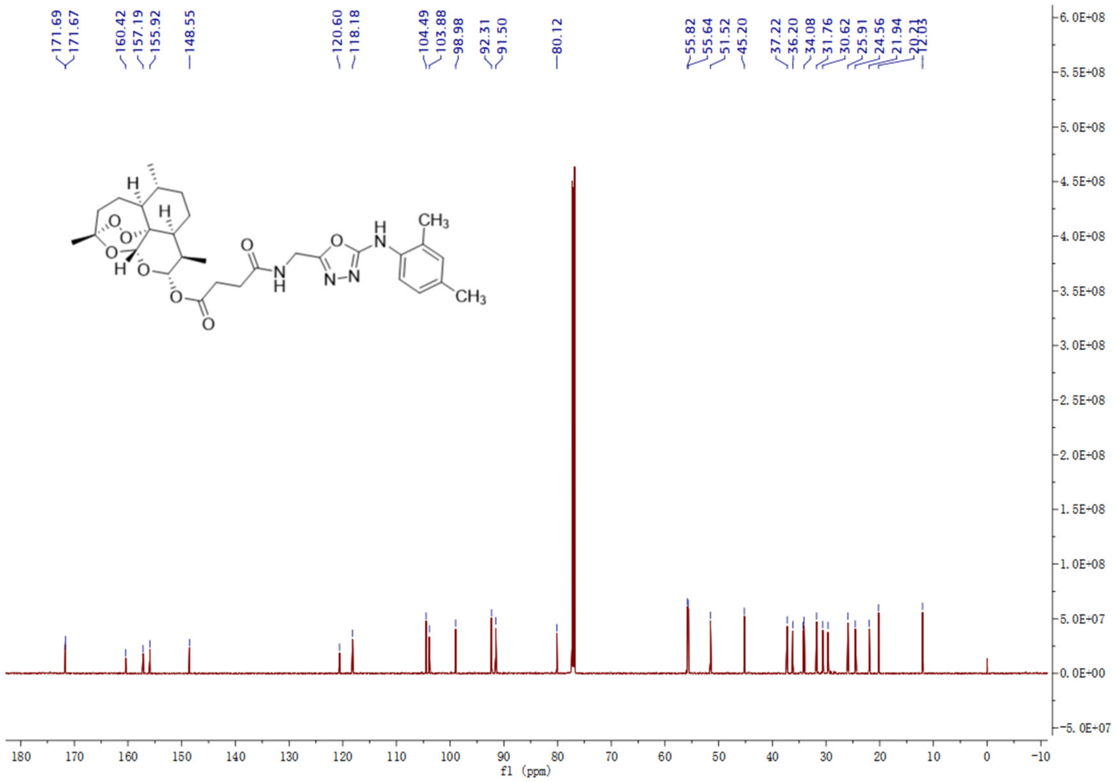

Figure S46. ^{13}C-NMR spectrum of compound **A15**

General Procedure of Intermediate 2a-2o.

The respective modified aniline (2 mmol, 1 eq) was dissolved in dry THF (20 mL). TEA (5 mmol, 2.5 eq) was added under inert atmosphere at 0°C. Then thiophosgene (2.4 mmol, 1.2 eq) was added dropwise over 20 min. The ice bath was removed and srirred for at least 2 h until TLC monitoring showed completion of reactions. The solvent was evaporated under reduced pressure, the residue was dissolved in EA and washed with saturated $NaHCO_3$ (2×30 mL) solution and brine (2×20 mL). The organic phase was dried over $MgSO_4$ and concentrated in vacuum. The desired Aryl isothiocyanates **2a**-**2o** were purified by flash column chromatography (silica gel, hexane).

4.1.2 General Procedure of Intermediate 3a-3o.

A solution of Intermediate **2a**-**2o** (1eq) and *t*-butyl (2-hydrazinyl-2-oxoethyl) carbamate (1eq) dissolved in dry THF was stirred overnight at room temperature. Then the solvent was evaporated under reduced pressure, the residue was dispersed in hexane and stirred for 2 h. The solid were filtered in vacuum and dried, using for next steps without any purification.

4.1.3 General Procedure of Intermediate 4a-4o.

EDCI (1.2eq) was added to a stirred solution of Intermediate **3a**-**3o** (1eq) in 20 mL DMSO. The reaction mixture was stirred at 60 °C for 5 h. Then the solution was poured into the mixture of ice and water and stirred vigorously for 2 h. The solid were filtered in vacuum and dried to afford desired intermediate **4a-4o**.

t-butyl((5-((4-(trifluoromethoxy)phenyl)amino)-1,3,4-oxadiazol-2-yl)methyl)carbamate (**4a**)

Yield 72.5%, white solid, ^{1}H NMR (600 MHz, DMSO-d_6) δ 10.71 (s, 1H), 7.64-7.66 (m, 2H), 7.54-7.56 (m, 1H), 7.35-7.36 (m, 2H), 4.28-4.29 (m, 2H), 1.40 (s, 9H). ESI-MS m/z 375.1 $[M+H]^+$.

t-butyl ((5-(p-tolylamino)-1,3,4-oxadiazol-2-yl)methyl)carbamate (**4b**)

Yield 75.4%, white solid, ^{1}H NMR (600 MHz, DMSO-d_6) δ 10.32 (s, 1H), 7.52-7.54 (m, 1H), 7.42-7.44 (m, 2H), 7.12-7.14 (m, 2H), 4.26-4.27 (m, 2H), 2.25 (s, 3H), 1.40 (s, 9H). ESI-MS m/z 305.1 $[M+H]^+$.

t-butyl ((5-((2-ethoxyphenyl)amino)-1,3,4-oxadiazol-2-yl)methyl)carbamate (**4c**)

Yield 68.9%, white solid, ^{1}H NMR (600 MHz, DMSO-d_6) δ 9.38 (s, 1H), 7.90 (m, 1H), 7.51-7.52 (m, 1H), 6.98-7.03 (m, 2H), 6.92-6.94 (m, 1H), 4.26-4.27 (m, 1H), 4.08 (q, *J* = 7.0 Hz, 2H), 1.40 (s, 9H), 4.08 (t, *J* = 7.0 Hz, 3H). ESI-MS m/z 335.1 $[M+H]^+$.

t-butyl ((5-((2,6-difluorophenyl)amino)-1,3,4-oxadiazol-2-yl)methyl)carbamate (**4d**)

Yield 67.3%, white solid, ^{1}H NMR (600 MHz, DMSO-d_6) δ 9.96 (s, 1H), 7.52 (m, 1H), 7.34-7.35 (m, 1H), 7.19-7.21 (m, 2H), 4.25-4.26 (m, 2H), 1.40 (s, 9H). ESI-MS m/z 327.1 $[M+H]^+$.

t-butyl((5-((4-(trifluoromethyl)phenyl)amino)-1,3,4-oxadiazol-2-yl)methyl)carbamate (**4e**)

Yield 75.4%, white solid, ^{1}H NMR (600 MHz, DMSO-d_6) δ 10.97 (s, 1H), 7.70-7.76 (m, 4H), 7.55-7.57 (m, 1H), 4.30-4.31 (m, 2H), 1.40 (s, 9H). ESI-MS m/z 359.1 $[M+H]^+$.

t-butyl ((5-((4-chlorophenyl)amino)-1,3,4-oxadiazol-2-yl)methyl)carbamate (**4f**)

Yield 65.9%, white solid, ^{1}H NMR (600 MHz, DMSO-d_6) δ 10.65 (s, 1H), 7.53-7.58 (m, 3H), 7.38-7.40 (m, 2H), 4.28-4.29 (m, 2H), 1.40 (s, 9H). ESI-MS m/z 325.1 $[M+H]^+$.

t-butyl ((5-((2,5-dibromophenyl)amino)-1,3,4-oxadiazol-2-yl)methyl)carbamate (**4g**)

Yield 58.3%, white solid, ^{1}H NMR (600 MHz, DMSO-d_6) δ 9.91 (s, 1H), 8.17 (s, 1H), 7.54-7.62 (m, 2H), 7.24-7.25 (m, 1H), 4.28-4.29 (m, 2H), 1.40 (s, 9H). ESI-MS m/z 447.1 $[M+H]^+$.

t-butyl ((5-((2,4-difluorophenyl)amino)-1,3,4-oxadiazol-2-yl)methyl)carbamate (**4h**)

Yield 68.1%, white solid, ^{1}H NMR (600 MHz, DMSO-d_6) δ 10.26 (s, 1H), 7.99-8.03 (m, 1H), 7.54-7.56 (m, 1H), 7.31-7.35 (m, 1H), 7.10-7.13 (m, 1H), 3.40 (s, 2H), 1.41 (s, 9H). ESI-MS m/z 327.1 $[M+H]^+$.

t-butyl ((5-((2-fluorophenyl)amino)-1,3,4-oxadiazol-2-yl)methyl)carbamate (**4i**)

Yield 66.9%, white solid, ^{1}H NMR (600 MHz, DMSO-d_6) δ 10.26 (s, 1H), 8.00-8.03 (m, 1H), 7.53-7.55 (m, 1H), 7.19-7.27 (m, 2H), 7.06-7.09 (m, 1H), 4,27-4.28 (m, 2H), 1.40 (s, 9H). ESI-MS m/z 309.1 $[M+H]^+$.

t-butyl((5-((4-(*t*-butyl)phenyl)amino)-1,3,4-oxadiazol-2-yl)methyl)carbamate (**4j**)

Yield 70.3%, white solid, ^{1}H NMR (600 MHz, DMSO-d_6) δ 10.33 (s, 1H), 7.52-7.54 (m, 1H), 7.45-7.46 (m, 2H), 7.33-7.35 (m, 2H), 4.26-4.27 (m, 2H), 1.40 (s, 9H), 1.26 (s, 9H). ESI-MS m/z 347.2 $[M+H]^+$.

t-butyl ((5-((4-bromophenyl)amino)-1,3,4-oxadiazol-2-yl)methyl)carbamate (**4k**)

Yield 65.1%, white solid, ^{1}H NMR (600 MHz, DMSO-d_6) δ 10.66 (s, 1H), 7.50-7.55 (m, 5H), 4.28-4.29 (m, 2H), 1.40 (s, 9H). ESI-MS m/z 369.0 $[M+H]^+$.

t-butyl ((5-(pyridin-3-ylamino)-1,3,4-oxadiazol-2-yl)methyl)carbamate (**4l**)

Yield 69.2%, white solid, ^{1}H NMR (600 MHz, DMSO-d_6) δ 10.76 (s, 1H), 8.71 (m, 1H), 8.21-8.22 (m, 1H), 8.02-8.03 (m, 1H), 7.55-7.57 (m, 1H), 7.37-7.39 (m, 1H), 4.29-4.30 (m, 2H), 1.40 (s, 9H). ESI-MS m/z 292.1 $[M+H]^+$.

t-butyl ((5-((3,5-dimethylphenyl)amino)-1,3,4-oxadiazol-2-yl)methyl)carbamate (**4m**)

Yield 71.5%, white solid, ^{1}H NMR (600 MHz, DMSO-d_6) δ 10.29 (s, 1H), 7.53-7.54 (m, 1H), 7.17 (m, 1H), 6.63 (m, 1H), 4.27-4.28 (m, 2H), 2.25 (s, 6H), 1.40 (s, 9H). ESI-MS m/z 319.1 $[M+H]^+$.

t-butyl ((5-((4-methoxyphenyl)amino)-1,3,4-oxadiazol-2-yl)methyl)carbamate (**4n**)

Yield 65.7%, white solid, ^{1}H NMR (600 MHz, DMSO-d_6) δ 10.21 (s, 1H), 7.51-7.53 (m, 1H), 7.45-7.47 (m, 2H), 6.91-6.92 (m, 2H), 4.25-4.26 (m, 2H), 3.72 (s, 3H), 1.40 (s, 9H). ESI-MS m/z 321.1 $[M+H]^+$.

t-butyl ((5-((2,4-dimethylphenyl)amino)-1,3,4-oxadiazol-2-yl)methyl)carbamate (**4o**)

Yield 71.3%, white solid, ^{1}H NMR (600 MHz, DMSO-d_6) δ 9.33 (s, 1H), 7.50-7.51 (m, 2H), 6.98-7.02 (m, 2H), 4.23-4.24 (m, 2H), 2.24 (s, 3H), 2.21 (s, 3H), 1.39 (s, 9H). ESI-MS m/z 319.2 $[M+H]^+$.

4.1.4 General Procedure of Intermediate 5a-5o.

A solution of Intermediate **4a-4o** (1eq) dissolved in 4M HCl/diox (20 mL) was stirred overnight at room temperature. Then the solvent was evaporated under reduced pressure, the residue was dissolved in MeOH, K_2CO_3 (0.8 g) was added and stirred for 5 minutes. The filtrate was collected and evaporated under reduced pressure to obtain intermediate **5a-5o** as soild, which was used for next steps without any purification.

4.1.5 General Procedure of hybrids A1-A15.

A solution of Intermediate **5a-5o** (1eq), artesunate (1eq), EDCI (1.2eq) and HOBt (1.2eq) dissolved in DMF (20 mL) was stirred for 5 h at room temperature until TLC monitoring showed completion of reactions. The solution was diluted with DCM and washed with water (2×20 mL) and brine (2×20 mL). The organic phase was dried over $MgSO_4$ and concentrated in vacuum. The desired products **A1-A15** were purified by flash column chromatography (silica gel).

Hybrid **A1**, white solid, yield 67.5%. m.p. 176.3-177.5°C, ^{1}H NMR (600MHz, $CDCl_3$) δ 7.58-7.60 (m, 2H), 7.30 (t, J = 5.6 Hz, 1H), 7.16-7.18 (m, 2H), 5.68 (d, J = 9.9 Hz, 1H), 5.40 (s, 1H), 4.60 (d, J = 5.7 Hz, 2H), 2.69-2.74 (m, 2H), 2.57-2.60 (m, 2H), 2.47-2.53 (m, 1H), 2.35 (td, J = 14.3, 3.9 Hz, 1H), 1.99-2.03 (m, 1H), 1.86-1.89 (m, 1H), 1.66-1.73 (m, 2H), 1.56-1.60 (m, 1H), 1.41-1.42 (m, 1H), 1.37 (s, 3H), 1.25-1.30 (m, 4H), 0.94 (d, J = 5.8 Hz, 3H), 0.77 (d, J = 7.1 Hz, 3H). ^{13}C NMR (150 MHz, $CDCl_3$) δ 172.25, 171.84, 160.20, 157.43, 144.26, 136.60, 122.06, 118.71, 104.64, 92.51, 91.59, 80.22, 51.47, 45.11, 37.22, 36.19, 34.38, 33.97, 31.72, 30.25, 29.37, 25.80, 24.51, 21.91, 20.15, 11.92. ESI-MS m/z 641.2 $[M+H]^+$, 663.2 $[M+Na]^+$.

Hybrid **A2**, white solid, yield 63.4%. m.p. 174.3-175.8°C, ^{1}H NMR (600MHz, $CDCl_3$) δ 7.39-7.41 (m, 2H), 7.11-7.13 (m, 2H), 7.08 (t, J = 5.5 Hz, 1H), 5.71 (d, J = 9.8 Hz, 1H), 5.39 (s, 1H), 4.57 (d, J = 5.6 Hz, 1H), 2.69-2.79 (m, 2H), 2.51-2.59 (m, 2H), 2.33-2.37 (m, 1H), 2.30 (s, 3H), 2.05 (s, 3H), 1.99-2.02 (m, 1H), 1.86-1.88 (m, 1H), 1.66-1.72 (m, 2H), 1.57-1.60 (m, 1H), 1.41-1.45 (m, 1H), 1.39 (s, 3H), 1.25-1.31 (m,

5H), 0.94 (d, J = 5.9 Hz, 3H), 0.79 (d, J = 7.1 Hz, 3H). ^{13}C NMR (150 MHz, $CDCl_3$) δ 171.96, 171.78, 160.57, 157.13, 135.19, 132.53, 129.75, 117.85, 104.56, 92.40, 91.53, 80.19, 60.42, 51.51, 45.16, 37.20, 36.22, 34.38, 34.04, 31.74, 30.41, 29.50, 25.86, 24.55, 21.91, 21.06, 20.74, 20.20, 14.20, 11.97. ESI-MS m/z 571.3 $[M+H]^+$, 593.2 $[M+Na]^+$.

Hybrid **A3**, white solid, yield 71.5%. m.p. 181.5-182.8°C, ^{1}H NMR (600MHz, $CDCl_3$) δ 8.07-8.09 (m, 1H), 6.99-7.00 (m, 2H), 6.85-6.88 (m, 2H), 5.76 (d, J = 9.9 Hz, 1H), 5.40 (s, 1H), 4.61 (t, J = 4.4 Hz, 2H), 4.13 (q, J = 6.9 Hz, 2H), 2.96 (s, 2H), 2.88 (s, 2H), 2.73-2.84 (m, 3H), 2.52-2.68 (m, 4H), 2.33-2.36 (m, 1H), 1.99-2.05 (m, 1H), 1.85-1.89 (m, 1H), 1.72-1.75 (m, 1H), 1.66-1.69 (m, 1H), 1.58-1.61 (m, 1H), 1.48 (t, J = 7.0 Hz, 3H), 1.41-1.43 (m, 4H), 1.23-1.37 (m, 4H), 0.94 (d, J = 6.0 Hz, 3H), 0.83 (d, J = 7.1 Hz, 3H). ^{13}C NMR (150 MHz, $CDCl_3$) δ 171.72, 171.66, 146.45, 126.87, 122.81, 121.10, 117.20, 110.82, 104.46, 92.28, 91.47, 80.10, 64.29, 51.48, 45.16, 37.18, 36.53, 36.16, 34.21, 34.03, 31.72, 31.46, 30.61, 29.63, 25.88, 24.52, 21.91, 20.18, 14.89, 12.01. ESI-MS m/z 601.3 $[M+H]^+$, 623.2 $[M+Na]^+$.

Hybrid **A4**, white solid, yield 73.4%. m.p. 179.4-180.9°C, ^{1}H NMR (600MHz, $CDCl_3$) δ 7.18-7.21 (m, 1H), 6.97-6.99 (m, 3H), 6.88 (d, J = 5.1 Hz, 1H), 5.75 (d, J = 9.8 Hz, 1H), 5.42 (s, 1H), 5.30 (s, 1H), 4.78 (s, 1H), 4.51-4.61 (m, 2H), 2.96 (s, 1H), 2.88 (s, 1H), 2.81 (s, 2H), 2.74-2.78 (m, 2H), 2.51-2.62 (m, 2H), 2.33-2.38 (m, 1H), 1.98-2.02 (m, 1H), 1.86-1.89 (m, 1H), 1.70-1.78 (m, 2H), 1.59-1.63 (m, 1H), 1.39 (s, 3H), 1.26-1.35 (m, 5H), 0.95-0.96 (m, 4H), 0.83 (d, J = 7.1 Hz, 3H). ^{13}C NMR (150 MHz, $CDCl_3$) δ 175.83, 171.77, 171.73, 162.62, 158.00, 157.97, 156.33, 156.31, 127.18, 127.12, 127.05, 126.96, 114.54, 112.12, 112.10, 111.97, 104.53, 96.38, 94.63, 92.31, 91.49, 91.18, 87.78, 80.13, 51.49, 45.17, 37.23, 36.17, 34.36, 34.04, 32.99, 31.72, 30.47, 29.54, 28.22, 25.81, 24.53, 21.93, 20.19, 11.98. ESI-MS m/z 593.3 $[M+H]^+$, 615.2 $[M+Na]^+$.

Hybrid **A5**, white solid, yield 68.9%. m.p. 173.3-175.1°C, ^{1}H NMR (600MHz, $CDCl_3$) δ 7.67-7.68 (m, 2H), 7.57-7.58 (m, 2H), 7.13 (t, J = 5.3 Hz, 1H), 5.69 (d, J = 9.9 Hz, 1H), 5.39 (s, 1H), 4.62 (d, J = 5.5 Hz, 2H), 2.70-2.77 (m, 2H), 2.54-2.63 (m, 2H), 2.50-2.53 (m, 1H), 2.33-2.38 (m, 2H), 2.00-2.03 (m, 2H), 1.88-1.90 (m, 2H), 1.72-1.74 (m, 4H), 1.69-1.71 (m, 5H), 1.57-1.61 (m, 1H), 1.37 (s, 3H), 0.78 (d, J = 7.1 Hz, 3H). ^{13}C NMR (150 MHz, $CDCl_3$) δ 172.19, 171.80, 167.75, 132.31, 130.94, 128.85, 126.52, 126.50,

117.24, 104.64, 92.51, 91.60, 80.22, 65.59, 51.48, 45.11, 37.23, 36.20, 34.40, 33.97, 31.73, 30.58, 30.33, 29.71, 29.43, 29.33, 25.82, 24.52, 21.92, 20.16, 19.20, 13.74, 11.94. ESI-MS m/z 625.3 $[M+H]^+$, 647.2 $[M+Na]^+$.

Hybrid **A6**, white solid, yield 63.7%. m.p. 177.8-178.9°C, ^{1}H NMR (600MHz, $CDCl_3$) δ 7.50-7.51 (m, 2H), 7.26-7.28 (m, 2H), 5.68 (d, *J* = 9.9 Hz, 1H), 5.39 (s, 1H), 4.58-4.59 (m, 2H), 2.71-2.74 (m, 2H), 2.56-2.59 (m, 2H), 2.49-2.53 (m, 1H), 2.32-2.38 (m, 1H), 1.99-2.03 (m, 1H), 1.86-1.90 (m, 1H), 1.67-1.73 (m, 2H), 1.57-1.60 (m, 1H), 1.37 (s, 3H), 1.23-1.31 (m, 5H), 0.95 (d, *J* = 5.8 Hz, 3H), 0.78 (d, *J* = 7.1 Hz, 3H). ^{13}C NMR (150 MHz, $CDCl_3$) δ 172.21, 171.81, 136.39, 129.17, 127.85, 118.98, 104.61, 92.46, 91.56, 80.19, 51.45, 45.09, 37.20, 36.18, 34.34, 33.98, 31.70, 30.26, 29.37, 25.80, 24.51, 21.90, 20.18, 11.94. ESI-MS m/z 591.2 $[M+H]^+$, 613.2 $[M+Na]^+$.

Hybrid **A7**, white solid, yield 68.1%. m.p. 182.3-184.1°C, ^{1}H NMR (600MHz, $CDCl_3$) δ 7.39-7.40 (m, 1H), 7.06-7.08 (m, 1H), 6.89 (m, 1H), 5.75 (d, *J* = 9.8 Hz, 1H), 5.38 (s, 1H), 4.60-4.68 (m, 2H), 2.88-2.96 (m, 3H), 2.76-2.84 (m, 2H), 2.56-2.68 (m, 3H), 2.32-2.37 (m, 1H), 1.99-2.03 (m, 1H), 1.85-1.89 (m, 1H), 1.67-1.76 (m, 2H), 1.58-1.62 (m, 1H), 1.40 (s, 3H), 1.22-1.36 (m, 5H), 0.95 (d, *J* = 5.8 Hz, 3H), 0.84 (d, *J* = 7.1 Hz, 3H). ^{13}C NMR (150 MHz, $CDCl_3$) δ 171.77, 171.66, 162.58, 159.04, 158.22, 136.27, 134.97, 133.35, 126.91, 122.48, 121.41, 109.92, 108.11, 104.52, 104.49, 104.36, 104.10, 103.81, 96.39, 94.64, 92.34, 91.48, 91.20, 89.67, 88.54, 87.76, 81.11, 80.36, 80.08, 78.95, 60.38, 52.49, 51.53, 51.48, 51.42, 45.43, 45.17, 44.43, 44.32, 37.47, 37.36, 37.23, 36.49, 36.36, 36.27, 36.22, 36.16, 34.74, 34.69, 34.19, 34.17, 34.09, 34.04, 31.70, 31.44, 30.77, 29.98, 29.78, 26.05, 25.94, 25.90, 25.85, 24.70, 24.66, 24.56, 24.52, 24.40, 22.12, 21.95, 21.04, 20.35, 20.27, 20.23, 20.18, 16.17, 14.18, 13.17, 12.70, 12.02. ESI-MS m/z 735.0 $[M+Na]^+$.

Hybrid **A8**, white solid, yield 75.4%. m.p. 179.0-181.3°C, ^{1}H NMR (600MHz, $CDCl_3$) δ 8.08-8.12 (m, 1H), 6.89-6.94 (m, 2H), 6.78 (t, *J* = 5.6 Hz, 1H), 5.74 (d, *J* = 9.9 Hz, 1H), 5.41 (s, 1H), 5.30 (s, 1H), 4.57-4.65 (m, 2H), 2.74-2.84 (m, 2H), 2.51-2.66 (m, 3H), 2.33-2.39 (m, 1H), 2.00-2.05 (m, 1H), 1.86-1.90 (m, 1H), 1.69-1.77 (m, 2H), 1.59-1.63 (m, 1H), 1.42-1.46 (m, 2H), 1.40 (s, 3H), 1.25-1.35 (m, 4H), 0.96 (d, *J* = 6.1 Hz, 3H), 0.83 (d, *J* = 7.1 Hz, 3H). ^{13}C NMR (150 MHz, $CDCl_3$) δ 171.81, 171.76, 159.73, 157.76, 120.60, 120.55, 111.57, 111.55, 111.43, 111.40, 104.53, 92.38, 91.52,

80.11, 51.49, 45.16, 37.23, 36.17, 34.30, 34.04, 31.71, 30.57, 29.57, 25.87, 24.53, 21.93, 20.18, 11.99. ESI-MS m/z 593.3 $[M+H]^+$, 615.2 $[M+Na]^+$.

Hybrid **A9**, white solid, yield 72.8%. m.p. 177.4-175.9°C, ^{1}H NMR (600MHz, $CDCl_3$) δ 8.14 (t, *J* = 8.1 Hz, 1H), 7.16-7.19 (m, 1H), 7.09-7.12 (m, 1H), 7.00-7.04 (m, 1H), 5.75 (d, *J* = 9.9 Hz, 1H), 5.41 (s, 1H), 4.58-4.66 (m, 2H), 2.88-2.96 (m, 3H), 2.72-2.85 (m, 2H), 2.52-2.66 (m, 3H), 2.33-2.38 (m, 1H), 2.00-2.03 (m, 1H), 1.86-1.90 (m, 1H), 1.68-1.76 (m, 2H), 1.58-1.62 (m, 1H), 1.40 (s, 3H), 1.24-1.35 (m, 4H), 0.95 (d, *J* = 5.9 Hz, 3H),0.83 (d, *J* = 7.1 Hz, 3H). ^{13}C NMR (150 MHz, $CDCl_3$) δ 171.77, 171.72, 124.90, 124.88, 123.41, 119.34, 115.04, 114.92, 104.51, 92.35, 91.50, 80.12, 51.48, 45.17, 37.22, 36.17, 34.32, 34.04, 31.71, 30.61, 29.61, 25.88, 24.53, 21.92, 20.19, 12.00. ESI-MS m/z 575.3 $[M+H]^+$, 597.2 $[M+Na]^+$.

Hybrid **A10**, white solid, yield 67.3%. m.p. 176.3-177.5°C, ^{1}H NMR (600MHz, $CDCl_3$) δ 7.44-7.46 (m, 2H), 7.33-7.35 (m, 2H), 7.09 (t, *J* = 5.6 Hz, 1H), 5.71 (d, *J* = 9.9 Hz, 1H), 5.40 (s, 1H), 4.58 (d, *J* = 5.7 Hz, 2H), 2.70-2.80 (m, 2H), 2.51-2.62 (m, 3H), 2.33-2.38 (m, 1H), 1.99-2.03 (m, 1H), 1.86-1.89 (m, 1H), 1.65-1.73 (m, 2H), 1.57-1.60 (m, 1H), 1.42-1.46 (m, 1H), 1.39 (s, 3H), 1.29 (s, 9H), 1.25-1.27 (m, 2H), 0.94 (d, *J* = 6.0 Hz, 3H), 0.79 (d, *J* = 7.1 Hz, 3H). ^{13}C NMR (150 MHz, $CDCl_3$) δ 171.94, 171.80, 160.67, 157.13, 145.83, 135.20, 126.05, 117.52, 104.56, 92.40, 91.52, 80.20, 51.50, 45.15, 37.19, 36.21, 34.37, 34.26, 34.02, 31.73, 31.40, 30.39, 29.51, 25.86, 24.53, 21.91, 20.20, 11.97. ESI-MS m/z 613.3 $[M+H]^+$, 635.3 $[M+Na]^+$.

Hybrid **A11**, white solid, yield 61.5%. m.p. 174.3-176.1°C, ^{1}H NMR (600MHz, $CDCl_3$) δ 7.41-7.45 (m, 4H), 7.20 (t, *J* = 5.1 Hz, 1H), 5.69 (d, *J* = 9.9 Hz, 1H), 5.39 (s, 1H), 4.59 (d, *J* = 5.5 Hz, 2H), 2.69-2.75 (m, 3H), 2.56-2.60 (m, 2H), 2.49-2.53 (m, 1H), 2.32-2.39 (m, 1H), 2.00-2.03 (m, 1H), 1.86-1.90 (m, 1H), 1.67-1.74 (m, 3H), 1.57-1.63 (m, 1H), 1.41-1.43 (m, 2H), 1.38 (s, 3H), 1.25-1.30 (m, 5H), 0.95 (d, *J* = 5.7 Hz, 3H), 0.78 (d, *J* = 7.1 Hz, 3H). ^{13}C NMR (150 MHz, $CDCl_3$) δ 172.21, 171.80, 136.90, 132.12, 119.30, 115.32, 104.60, 104.51, 92.45, 92.30, 91.54, 91.53, 80.19, 80.13, 60.42, 51.52, 51.46, 45.18, 45.10, 37.20, 36.18, 34.33, 34.04, 33.98, 31.78, 31.70, 30.30, 29.41, 29.09, 28.56, 25.89, 25.81, 24.55, 24.51, 21.95, 21.90, 20.19, 14.19, 12.00, 11.95. ESI-MS m/z 635.2 $[M+H]^+$, 657.2 $[M+Na]^+$.

Hybrid **A12**, white solid, yield 71.2%. m.p. 175.7-177.2℃, ^{1}H NMR (600MHz, DMSO-d_6) δ 8.68 (m, 1H), 8.16-8.17 (m, 1H), 8.00-8.01 (m, 1H), 7.34-7.36 (m, 1H), 5.38 (s, 1H), 4.60 (d, J = 9.0 Hz, 1H), 2.31-2.45 (m, 1H), 2.15-2.20 (m, 1H), 2.07-2.11 (m, 1H), 1.97-1.99 (m, 1H), 1.78-1.85 (m, 2H), 1.63-1.67 (m, 1H), 1.55-1.60 (m, 2H), 1.33-1.41 (m, 6H), 1.28 (s, 3H), 1.12-1.17 (m, 2H), 0.88 (d, J = 6.4 Hz, 3H), 0.83 (d, J = 7.4 Hz, 2H), 0.78 (d, J = 7.2 Hz, 3H). ^{13}C NMR (151 MHz, DMSO-d_6) δ 177.22, 160.86, 155.25, 142.77, 139.77, 124.18, 124.10, 103.63, 103.53, 95.04, 93.69, 90.49, 87.12, 81.07, 80.64, 51.78, 45.54, 36.78, 36.54, 34.37, 34.32, 32.96, 28.59, 26.13, 24.81, 21.75, 20.59, 13.31. ESI-MS m/z 569.2 $[M+H]^+$, 591.3 $[M+Na]^+$.

Hybrid **A13**, white solid, yield 59.7%. m.p. 178.5-179.9℃, ^{1}H NMR (600MHz, $CDCl_3$) δ 7.15 (m, 2H), 7.09 (t, J = 5.5 Hz, 1H), 6.68 (s, 1H), 5.69 (d, J = 9.8 Hz, 1H), 5.38 (s, 1H), 4.58 (d, J = 5.6 Hz, 1H), 2.70-2.80 (m, 2H), 2.51-2.60 (m, 3H), 2.32-2.38 (m, 1H), 2.29 (s, 6H), 1.99-2.03 (m, 1H), 1.85-1.88 (m, 1H), 1.65-1.72 (m, 2H), 1.56-1.59 (m, 1H), 1.41-1.44 (m, 1H), 1.39 (s, 3H), 1.22-1.32 (m, 4H), 0.94 (d, J = 5.7 Hz, 3H), 0.79 (d, J = 7.1 Hz, 3H). ^{13}C NMR (150 MHz, $CDCl_3$) δ 172.00, 171.81, 160.42, 157.15, 139.02, 137.54, 124.79, 115.46, 104.55, 92.42, 91.52, 80.18, 51.49, 45.15, 37.18, 36.21, 34.36, 34.04, 31.72, 30.40, 29.50, 25.85, 24.53, 21.90, 21.44, 20.19, 14.20, 11.95. ESI-MS m/z 585.3 $[M+H]^+$, 607.3 $[M+Na]^+$.

Hybrid **A14**, white solid, yield 76.2%. m.p. 175.7-176.9℃, ^{1}H NMR (600MHz, $CDCl_3$) δ 7.42-7.43 (m, 2H), 7.14 (t, J = 5.5 Hz, 1H), 6.85-6.87 (m, 2H), 5.71 (d, J = 9.8 Hz, 1H), 5.40 (s, 1H), 4.56 (d, J = 5.6 Hz, 1H), 3.77 (s, 3H), 2.69-2.79 (m, 3H), 2.50-2.59 (m, 3H), 2.33-2.38 (m, 1H), 2.00-2.02 (m, 1H), 1.86-1.89 (m, 1H), 1.66-1.73 (m, 2H), 1.57-1.60 (m, 1H), 1.41-1.45 (m, 1H), 1.39 (s, 3H), 1.24-1.33 (m, 5H), 0.94 (d, J = 5.8 Hz, 3H), 0.80 (d, J = 7.1 Hz, 3H). ^{13}C NMR (150 MHz, $CDCl_3$) δ 171.99, 171.80, 160.89, 157.09, 155.60, 131.07, 119.62, 114.47, 104.57, 92.40, 91.53, 80.20, 55.54, 51.50, 45.16, 37.19, 36.21, 34.35, 34.04, 31.73, 30.36, 29.48, 25.84, 24.55, 21.91, 20.19, 11.98. ESI-MS m/z 587.3 $[M+H]^+$, 609.3 $[M+Na]^+$.

Hybrid **A15**, white solid, yield 71.9%. m.p. 181.3-182.5℃, ^{1}H NMR (600MHz, $CDCl_3$) δ 7.94-7.95 (m, 1H), 6.73 (t, J = 5.3 Hz, 1H), 6.50-6.52 (m, 2H), 5.76 (d, J = 9.8 Hz, 1H), 5.40 (s, 1H), 4.59 (d, J = 5.6 Hz, 1H), 3.87 (s, 3H), 3.80 (s, 3H), 2.73-2.84 (m, 2H), 2.61-2.66 (m, 1H), 2.52-2.58 (m, 2H), 2.33-2.38 (m, 1H), 2.00-2.03 (m, 1H), 1.86-1.89 (m, 1H), 1.67-1.76 (m, 3H), 1.58-1.62 (m, 1H), 1.43-1.46 (m, 1H), 1.41 (s, 3H),

1.23-1.37 (m, 5H), 0.95 (d, *J* = 5.9 Hz, 3H), 0.84 (d, *J* = 7.1 Hz, 3H). ^{13}C NMR (150 MHz, $CDCl_3$) δ 171.69, 171.67, 160.42, 157.19, 155.92, 148.55, 120.60, 118.18, 104.49, 103.88, 98.98, 92.31, 91.50, 80.12, 55.82, 55.64, 51.52, 45.20, 37.22, 36.20, 34.27, 34.08, 31.76, 30.62, 29.64, 25.91, 24.56, 21.94, 20.21, 12.03. ESI-MS m/z 617.2 $[M+H]^+$, 639.2 $[M+Na]^+$.

Inhibition rate curve of some compounds (The inhibition rate is a percentage, and the unit of concentration is μM)

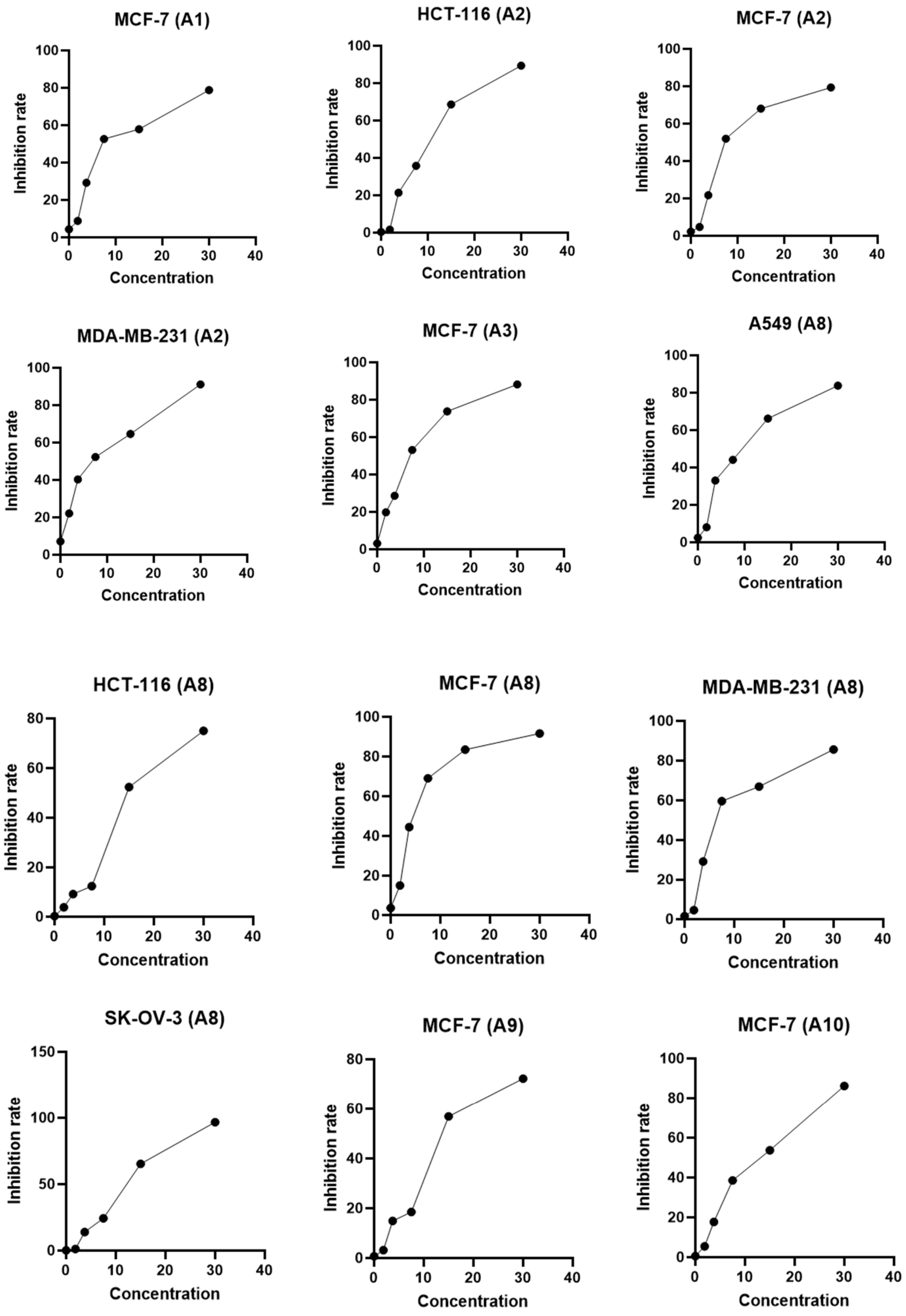
MCF-7 (A1)
HCT-116 (A2)
MCF-7 (A2)
MDA-MB-231 (A2)
MCF-7 (A3)
A549 (A8)
HCT-116 (A8)
MCF-7 (A8)
MDA-MB-231 (A8)
SK-OV-3 (A8)
MCF-7 (A9)
MCF-7 (A10)
Inhibition rate
Concentration

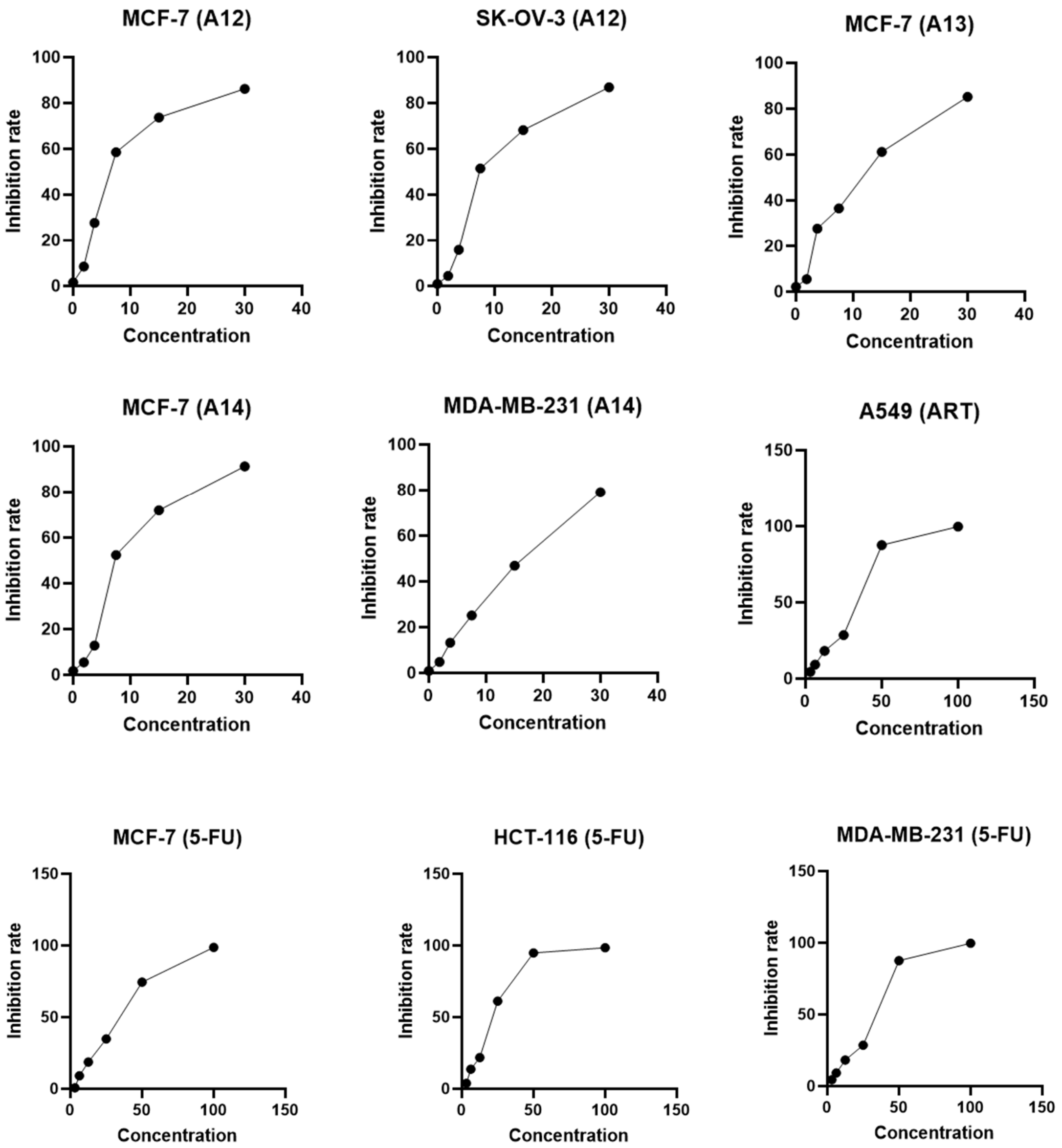
MCF-7 (A12)
SK-OV-3 (A12)
MCF-7 (A13)
MCF-7 (A14)
MDA-MB-231 (A14)
A549 (ART)
MCF-7 (5-FU)
HCT-116 (5-FU)
MDA-MB-231 (5-FU)
Inhibition rate
Concentration
